# Slab remnants beneath the Myanmar terrane evidencing double subduction of the Neo-Tethyan Ocean

**DOI:** 10.1126/sciadv.abo1027

**Published:** 2022-08-26

**Authors:** Shun Yang, Xiaofeng Liang, Mingming Jiang, Lin Chen, Yumei He, Chit Thet Mon, Guangbing Hou, Myo Thant, Kyaing Sein, Bo Wan

**Affiliations:** ^1^ Key Laboratory of Earth and Planetary Physics, Institute of Geology and Geophysics, Chinese Academy of Sciences (CAS), Beijing 100029, China.; ^2^ University of Chinese Academy of Sciences, Beijing 100049, China.; ^3^ State Key Laboratory of Lithospheric Evolution, Institute of Geology and Geophysics, CAS, Beijing 100029, China.; ^4^ Department of Geology, Dagon University, Yangon, Myanmar.; ^5^ Department of Geology, Yangon University, Yangon, Myanmar.; ^6^ Myanmar Earthquake Committee, Yangon, Myanmar.; ^7^ Myanmar Geosciences Society, Yangon, Myanmar.

## Abstract

Closure of the Neo-Tethyan Ocean is one of the most significant tectonic events of the Cenozoic, forming the longest continental collision belt on Earth and influencing global climate and biodiversity. However, whether late Mesozoic subduction of the Neo-Tethyan Ocean occurred along one single or a double subduction system remains controversial. Here, upper mantle imaging from seismic tomography and waveform modeling in the Myanmar region reveals two prominent, parallel, slab-like structures with high seismic velocities that trend to the north-south and dip to the east. The western high-velocity zone has been observed previously and represents the modern subducting slab. The eastern zone has not been previously reported and exhibits high-velocity anomalies of 1.0 to 2.5% to a depth of ~300 km. This zone likely represents a remnant of another Neo-Tethyan oceanic slab that subducted ~40 million years ago. Double subduction of the Neo-Tethyan Ocean during the late Mesozoic to early Cenozoic requires reevaluation of previous tectonic models.

## Teaser

Seismic evidence supports the Neo-Tethyan double subduction model.

## INTRODUCTION

Subduction of the Neo-Tethyan Ocean led to collision between India and Asia (*1*, *2*). However, before collision, convergence between India and Asia was anomalously fast and exceeded modern average subduction rates by a factor of 2 (*3*, *4*). Two models have been proposed to explain this anomalous speed: a rapid single subduction system due to thinned Indian lithosphere (*5*) or a double subduction system involving a second, northward-subducting oceanic slab (*6*). Although the double subduction model is partly supported by geological and paleomagnetic evidence as well as model-based arguments (*6*–*8*), no compelling seismic evidence of double subducted slabs in the present upper mantle has been reported along the entire Himalaya and adjacent regions. This evidence would provide major support for the double subduction model (*9*). However, strong postcollisional deformation or slab loss after the initial collision of India and Asia could readily obscure a second slab.

**Fig. 1.**
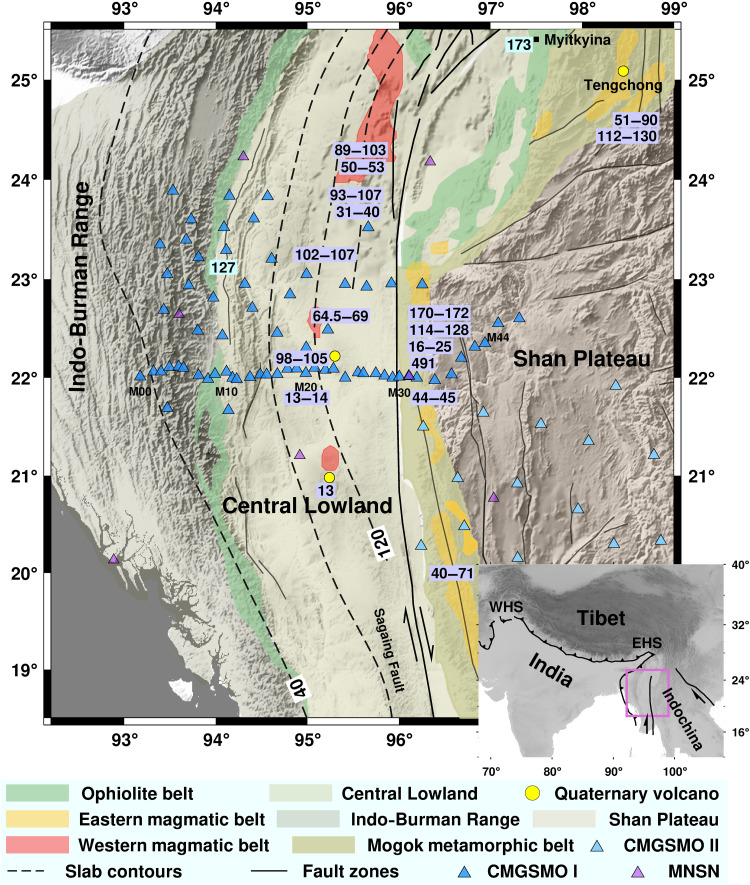
Overview map of the study area with the major geological units. After Licht *et al.* (*69*) and Wang *et al.* (*70*). The black dashed lines indicate depth to the top of the (western) subducting slab (*71*). The numbers in cyan boxes represent ages of ophiolites (ages in millions of years) (*18*). The numbers in purple boxes represent ages of magmatic rocks (ages in millions of years) (*19*, *24*, *26*–*33*, *72*). Triangles show locations of seismic stations. Note the dense east-west array at ~22°N latitude (stations M00 to M44). The bottom-right inset represents the location of our study area (magenta rectangle) in the Indian-Asian collisional system. CMGSMO, China-Myanmar Geophysical Survey in the Myanmar Orogen; MNSN, Myanmar National Seismic Network; EHS, eastern Himalayan syntaxis; WHS, western Himalayan syntaxis.

Continental subduction in the central and western parts of the Himalayan orogen extends to a depth of ~300 to 400 km, and the surface is characterized by high mountain ranges (*10*, *11*). In this intensely reworked area, severe collision and shortening lead to slab break-offs and/or superposition and greatly weaken the traceability of slab structures, thus geophysics-based identification of a second slab would be especially difficult. In contrast, with its lateral position to the collision, the Myanmar sector may not have experienced severe tectonic reworking. Unlike the central and western sectors, it features low relief mountains and basins (Fig. 1), and the presently subducting continental plate penetrates to only a depth of ~100 km (*12*). Previous seismic tomography studies image slab-like high-velocity bodies in the mantle beneath Myanmar (*13*–*15*), providing possible evidence for Neo-Tethyan subduction. However, a high-resolution image of seismic velocity structure for the upper mantle has been missing, mainly because seismic stations have been sparse.

The Myanmar region occupies the eastern end of the Indian-Asian collisional system (*16*). Two north-south (N-S) linearly distributed ophiolite belts separate Myanmar into three tectonic units: the Shan Plateau in the east, the Central Lowland in the middle, and the Indo-Burman Range in the west (Fig. 1). The Shan Plateau belongs to the Sibumasu Block, which marks the southern continental margin of Asia (*17*). A Middle Jurassic [~173 million years (Ma) ago] ophiolite separates the Shan Plateau and Central Lowland (*18*). Magmatic rocks with subduction-related geochemical signatures occur along the western margin of the Shan Plateau (i.e., the eastern magmatic belt) and have ages of ~170, 128 to 120, and 90 to 45 Ma (*19*), indicating long-lived subduction. High-grade metamorphic rocks along the boundary of the Shan Plateau and Central Lowland may reflect Indian oblique convergence with peak collisional metamorphism commencing ~45 Ma (*20*–*22*). Both the Central Lowland and Indo-Burman Range are associated with Neo-Tethyan subduction (*23*, *24*). The Central Lowland is defined as part of the West Burma Block (*25*). A magmatic belt straddles the middle of the Central Lowland (i.e., the western magmatic belt), which has ages of 107 to 89, 69 to 31, and <14 Ma (*19*, *24*, *26*–*33*). The boundary between the Central Lowland and the Indo-Burman Range is mainly an Early Cretaceous (~127 Ma) ophiolite (*18*). The Indo-Burman Range represents a forearc accretionary prism attached to the West Burma Block (*34*). The collision-induced extrusion in Myanmar was initiated in Oligocene (~27 Ma) and roughly along the western margin of the Shan Plateau, where the eastern magmatic belt was located (*16*).

Here, we use data from novel seismic arrays associated with the China-Myanmar Geophysical Survey in the Myanmar Orogen (CMGSMO) (*12*, *35*) to investigate the upper mantle structure in the Myanmar region. By compiling teleseismic finite-frequency tomography, two-dimensional (2D) forward waveform modeling, and geodynamic numerical modeling, we reveal a clear image of subducted slabs that implies double subduction of the Neo-Tethyan Ocean.

## RESULTS

### Seismic tomography

The finite-frequency body-wave tomography method (*36*) was used to construct upper mantle images beneath Myanmar and surrounding regions. The core data for tomographic interpretation were collected from the CMGSMO project, which deployed the first dense seismic array in Myanmar (Fig. 1) (*35*). The CMGSMO arrays (CMGSMO I, 2016–2018, and CMGSMO II, 2018–2020) used in this study contain 84 stations equipped with broadband seismometers and distributed in Central Myanmar (20.1°N to 23.9°N and 93.1°E to 98.9°E) (see Materials and Methods; Fig. 1). Seismic data collected from surrounding networks were supplemented to enlarge the observational aperture and improve the resolution in the deep upper mantle (see Materials and Methods; fig. S1).

**Fig. 2.**
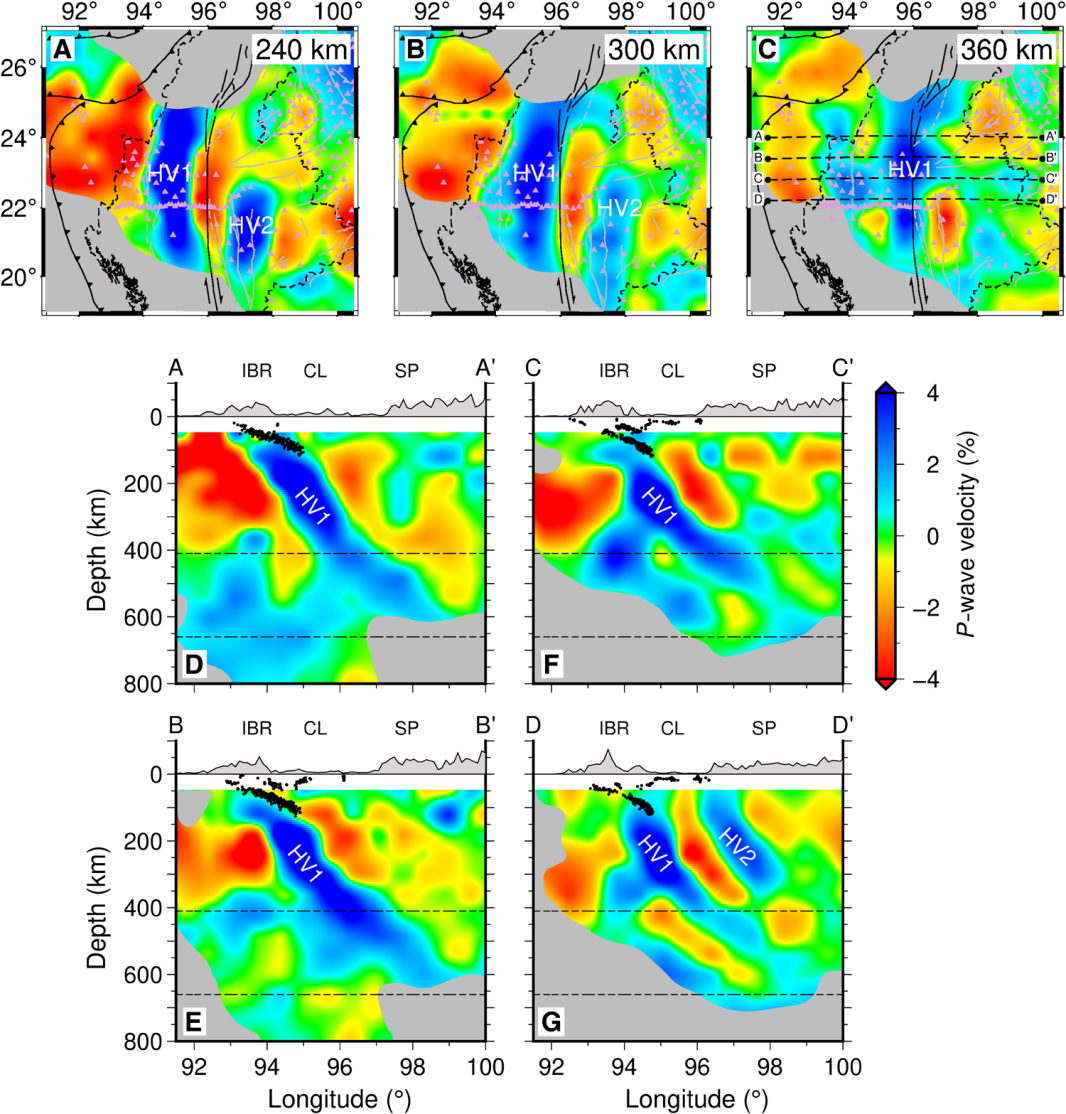
Tomographic model of *P*-wave velocity. (**A to C**) Horizontal slices of *P*-wave velocity beneath our study region at depths of 240, 300, and 360 km. The gray and black solid lines represent geological structures. The light purple triangles represent the distribution of seismic stations. The thick black dashed lines AA′, BB′, CC′, and DD′ in (C) mark the locations of the four cross sections presented in (D) to (G) and figs. S2 to S9. (**D** to **G**) Vertical slices of final *P*-wave velocity beneath our study region. Black dots mark the locations of earthquakes (*35*). Black dashed lines depict the 410- and 660-km discontinuities. The images are clipped on the basis of the intensity of *P*-wave travel-time sensitivity kernel (fig. S2). IBR, Indo-Burman Range; CL, Central Lowland; SP, Shan Plateau.

Our tomographic results prominently feature two subparallel, N-S–trending, east-dipping, seismic high-velocity anomalies (HV1 and HV2) that stretch along the strike of the main tectonic units (Fig. 2). The western anomaly (HV1) has a thickness of ~100 km and high-velocity anomalies of 2.0 to 4.0% beneath the West Burma Block. The distal end of HV1 reaches depths of 400 to 600 km (Fig. 2). The eastern anomaly (HV2) is clearly observed beneath the Shan Plateau between latitudes of 19.0° and 23.0°N. It can be continuously traced southward to the resolution limitation of tomography and might extend further south, while a low-velocity body below the Tengchong volcanic field blurs its northward extension. HV2 has a thickness of ~100 km and high-velocity anomalies of 1.0 to 2.5%. It extends to a depth of 300 km (Fig. 2). The spatial extent, shape, and seismic velocity anomaly of HV2 demonstrate that it is a regional-scale fabric and should have substantial tectonic implications.

The seismic data from the dense CMGSMO arrays, as well as the supplementary dataset surrounding the study area, correctly constrain their shapes (see Materials and Methods; figs. S2 and S3). Both HV1 and HV2 fall into the areas with good tomographic resolution according to the 3D data coverage (figs. S2 and S3). Although previous tomographic images (*13*, *15*) indicated high-velocity anomalies at the location of HV1 (fig. S4), our high-resolution images show that HV1 closely resembles a subducted slab because its thickness remains nearly unchanged in the upper mantle. HV2 is invisible in previous studies (*13*, *15*) but distinctly resolved by our data. Synthetic tests with specified anomaly structures further verify the reliability of the existence and geometries of HV1 and HV2 (see Materials and Methods; figs. S5 to S9).

### Waveform modeling

**Fig. 3.**
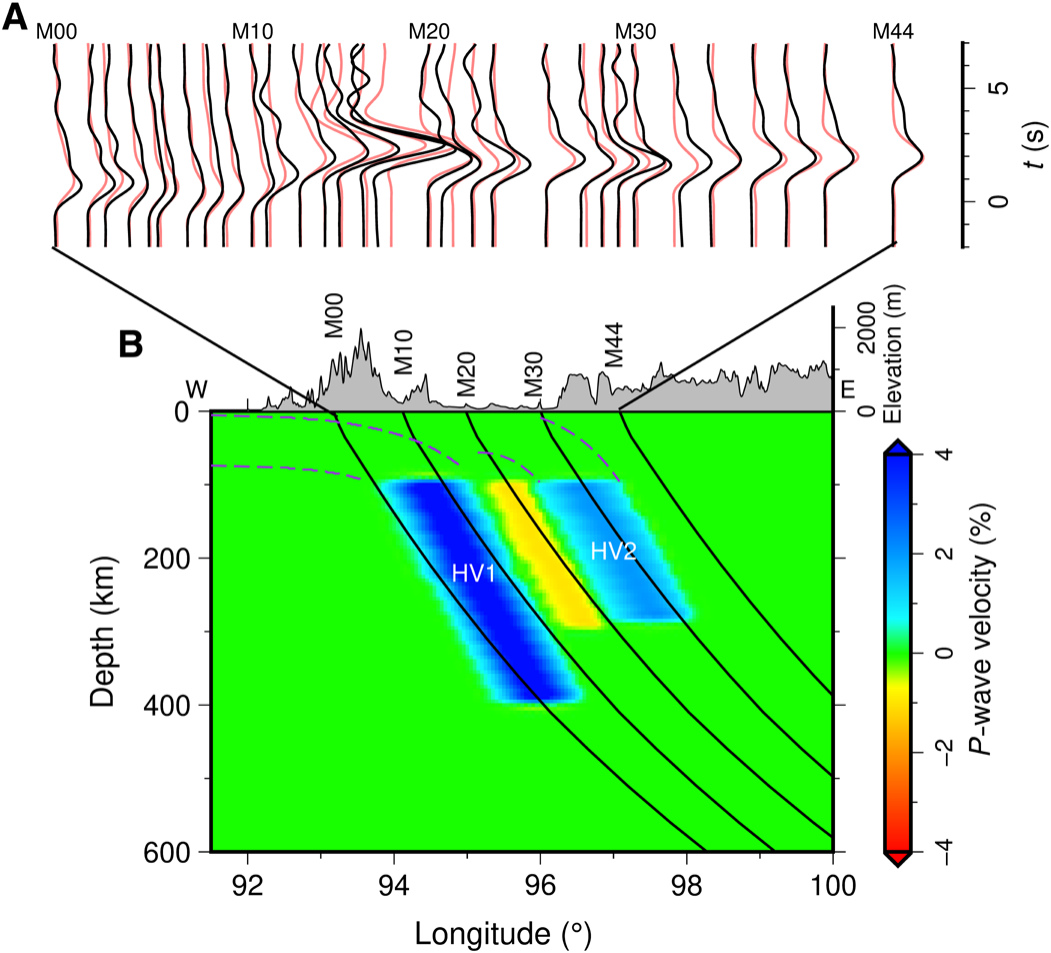
2D waveform modeling of event 2017/09/07. (**A**) Comparison between observed (black lines) and synthetic (red lines) vertical displacements generated by a model composing the crust structure (*41*) and the upper mantle model shown in (B). (**B**) The best-fitting model and the ray paths of *P* waves at the receiver side. The possible extensions of two slabs (HV1 and HV2) to a shallow depth are marked by purple dashed lines. The synthetic HV1 has a velocity perturbation of 4.5%, a thickness of 130 km, and extends to a depth of 400 km, and HV2 has a velocity perturbation of 2.0%, a thickness of 120 km, and reaches to a depth of 300 km. The ray paths are computed on the basis of the IASP91 reference Earth model and nearly parallel to HV1 and HV2 in the upper mantle.

Seismic tomography usually yields an average solution that best satisfies a wide range of travel time data but does not account for details of observed seismic waveforms. Waveform modeling is a powerful tool to refine mantle structural imaging (*37*, *38*), complementing the seismic tomographic results. In the waveform modeling method, systematic waveform distortions in the observed seismograms are identified and compared to synthetic waveforms from a series of plausible velocity models in an effort to select a best-fitting velocity model. We use waveform forward modeling to further validate the two parallel, east-dipping, high-velocity anomalies that we inferred from tomography. We analyzed seismic waveforms in detail along our dense, east-west–trending, linear subarray (stations M00 to M44 with station spacings of 10 to 15 km; Fig. 1). Apparent *P* waveform distortions are variably observed among different stations from several teleseismic events whose ray paths parallel the dip of HV1 and HV2. Stations M00 to M10 show anomalously broadened, low-amplitude waveforms. From stations M12 to M18, waveforms gradually manifest delayed arrivals with narrow high amplitude. Farther to the east, waveforms with decreasing amplitudes and earlier arrivals can be traced from stations M20 to M22, while relatively normal waveforms are observed between stations M23 and M44 (Fig. 3 and fig. S10). We exclude other possibilities contributing to the waveform distortions and conduct 2D forward waveform modeling (see Materials and Methods) (*39*) to examine the influence of receiver-side upper mantle heterogeneities that we imaged beneath the linear subarray (latitude of ~22.2°N; Fig. 2G).

A synthetic model can match the observations well only if it comprises two separate high-velocity anomalies, mimicking HV1 and HV2 (Fig. 3A). At stations M00 to M10, conjoined *P*-wave arrivals (propagating separately through HV1 and outside of HV1) produce broad, low-amplitude waveforms. Further to the east, both HV1 and HV2 act as waveguides (see Materials and Methods; fig. S11) to gradually narrow and amplify waveforms until station M18. The absence of HV2 would lead to an amplitude decrease at stations M17 and M18 and a distinct arrival delay at stations M23 to M32 comparing with the observed waveforms (fig. S12). A series of waveform modeling tests suggest that HV1 has optimal velocity perturbations of 3.6 to 5.1%, thicknesses of 80 to 150 km, and penetration depths of 360 to 480 km. The corresponding parameters of HV2 are 1.2 to 2.7%, 60 to 120 km, and 230 to 390 km, respectively (see Materials and Methods; fig. S13).

## DISCUSSION

### Double subduction of the Neo-Tethyan Ocean

On the basis of the tomographic and waveform modeling results, we observe two parallel high-velocity bodies HV1 and HV2 with similar widths of ~100 km. In the west, an east-dipping Benioff seismicity zone marked by earthquakes along and within the subducted slab ceases at a shallow depth of ~100 km and appears to connect the top of HV1 (Fig. 2) (*12*). The earthquakes are interpreted to occur mainly in the crust of the subducted Indian continental plate (*12*). We interpret HV1 as the subducting Indian oceanic slab, connecting to the Indian continental plate at a shallow depth (*12*, *40*, *41*). The western magmatic belt, active from the middle Cretaceous to the late Cenozoic (*24*, *28*, *42*), and the related western ophiolite belt (*18*) logically mark the long subduction history spanning from the Neo-Tethyan Ocean to the present Indian oceanic slab. Plate reconstruction based on recent paleomagnetic data in Myanmar proposed that the Neo-Tethyan Ocean maintained a width of ~2000 km at ~95 Ma and closely followed by subduction of the Indian plate in the late Eocene (*8*). The length of HV1 along the dip is ~400 to 700 km (Fig. 2, D to G), much shorter than the expectation for the continuously subducted Neo-Tethyan and Indian oceanic slabs (*8*). Most of the subducted slab likely detached and sank into the deep mantle to the southwest of the current slab (*43*).

HV2 in the east is spatially separated from HV1 and probably reflects a different tectonic origin. Upper mantle high-velocity anomalies with characteristics similar to HV2 may be attributed to either subducted lithosphere or delaminated continental lithosphere. However, lithospheric delamination is usually characterized by widespread and short-term volcanism in addition to the magmatic arc (*44*, *45*). HV2 projects to the surface close to the transition zone between the Central Lowland and Shan Plateau (Fig. 2). The corresponding area and surrounding regions lack recent massive igneous activity, and the Jurassic-Cenozoic magmatism is generally distributed linearly along an N-S direction (Fig. 1) (*19*). Thus, slab-like HV2 more likely represents subducted lithosphere. Considering that upper mantle velocity perturbations are primarily attributed to temperature effects (*46*), a longer stagnant time of a slab in the mantle means a longer duration of heat exchange between a cold (high-velocity) slab and the ambient mantle. As a result, the slab would manifest a relatively high temperature and, therefore, a smaller high-velocity anomaly. A relatively small high-velocity perturbation of HV2 thus implies an older subduction age comparing with HV1.

On the basis of magmatic, ophiolitic, and metamorphic rocks related to closure of the Meso-Tethyan Ocean, previous studies have proposed that subduction beneath the Shan Plateau was active only from the Middle Triassic to the Early Cretaceous (*18*, *21*, *24*, *26*, *47*). However, a subducted slab is unlikely to be stalled in the upper mantle for more than 120 Ma due to its higher density relative to the surrounding mantle (*48*). In addition, thermal diffusion alone would erase any distinct high-velocity seismic anomalies after such a long period (i.e., a slab would not be observable seismically; see Materials and Methods), while magmas with subduction geochemistry in the region are as young as ~45 Ma (*19*). Accordingly, we interpret the HV2 seismic anomaly as a (Neo-)Tethyan slab subducted in the early Cenozoic rather than a Meso-Tethyan slab subducted at 120 Ma.

**Fig. 4.**
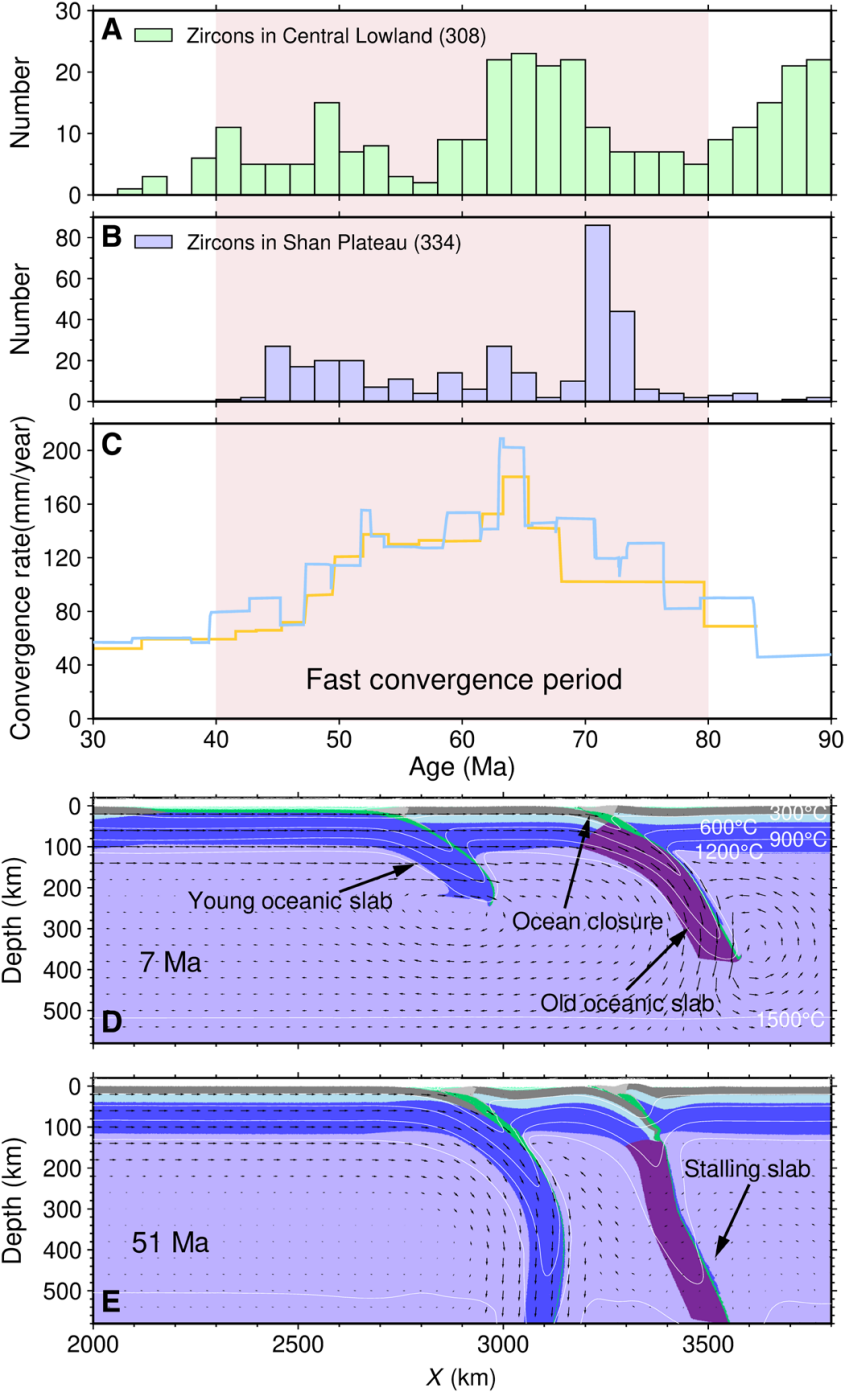
Plot of zircon age, India-Eurasia convergence rates (mm/year), and numerical modeling. (**A** and **B**) Compilation of zircons from the western magmatic belt and sedimentary rocks in the Central Lowland and eastern magmatic belt in the Shan Plateau. (**C**) India-Eurasia convergence rates (mm/year) from Cande and Stegman (*3*) (orange line) and White and Lister (*4*) (blue line). The pink shaded area marks a period of fast convergence between the Indian and Eurasian plates. (**D** and **E**) Numerical modeling of double subduction, showing the long-term stalling of an old and dense oceanic slab with the aid of a close young subparallel subducting slab. Black arrows indicate velocities. White lines represent isotherms with an interval of 300°C. The time is counted from the start of the model.

Recent paleomagnetic data reveal an extra branch of the Neo-Tethyan Ocean between the West Burma Block and Sibumasu Block that closed at ~40 Ma (*8*). Collision-related metamorphism that initiated ~45 Ma (*22*) and subduction-related magmatism from the Late Cretaceous to early Eocene (90 to 45 Ma) (*19*) further support the closure of an ocean at ~45 Ma along the western margin of the Shan Plateau. In that context, HV2 logically represents a remnant oceanic subducted slab attached to the eastern margin of the West Burma Block and preserved in the present upper mantle since the Eocene. Zircons for the western magmatic belt and sedimentary rocks in the Central Lowland and eastern magmatic belt in the Shan Plateau show widely overlapping age groups between 40 and 80 Ma (Fig. 4, A and B, and table S1) (*24*, *26*, *29*, *30*, *42*, *49*, *50*). These zircons are interpreted to have formed during subduction-related magmatism (*19*, *24*, *42*). Accordingly, they support coeval double subduction along with the West Burma Block and the Sibumasu Block between ~80 and ~40 Ma, corresponding to the period of fast convergence between the Indian and Eurasian plates (Fig. 4, A to C).

Latter collision-induced extrusion would not change our implication for two reasons. First, the spatial relationship of the two slabs represented by HV1 and HV2 would be modified slightly as they fell into the same tectonic regime during the extrusion (*16*). Second, the extrusion would merely elongate the eastern magmatic belt along strike, and most of the geochemical signals would be still confined within the belt. Therefore, we can link the HV2 to the eastern magmatic belt by integrating all the signals along strike and identifying their spatial coherence along the 2D transecting profile.

If double subduction explains rapid convergence between the Indian and Eurasian continents (*6*), diverse evidence should exist across vast regions of the Neo-Tethyan Ocean. Many recent geological observations including Mesozoic intraoceanic arcs (*7*) and suprasubduction ophiolites (*51*, *52*) as well as paleomagnetic arguments (*8*, *53*, *54*) along the convergent boundary of the Neo-Tethyan Ocean have been taken as the evidence of double subduction. Tomographic images of two detached high-velocity anomalies below a depth of ~1000 km beneath India (*9*) might be naturally interpreted as slab remnants of double subduction rather than one subducting slab, although there is no solid evidence to further reveal the origination of two detached anomalies. The presence, locations, and shapes of HV1 (the western slab) and HV2 (the eastern slab) in the upper mantle beneath Myanmar provide additional strong seismic evidence for double subduction of the Neo-Tethyan Ocean.

### Numerical modeling of double subduction

To examine how a double subduction system can be preserved in the upper mantle, we constructed geodynamic models in which two subducting oceanic slabs are separated by a microcontinental block (see Materials and Methods; fig. S14). To enable synchronous subduction of the two slabs, the eastern slab is set to be older and denser than the western one, as was also assumed by Jagoutz *et al.* (*6*). Our simulation shows that when the initial width of the microcontinental block between two subduction zones is 550 km, the corner flow caused by the ongoing subduction of the western slab provides vigorous resistance against the bending and sinking of the dense eastern slab (Fig. 4, D and E, and fig. S15). The dense eastern slab would be preserved in the upper mantle over 40 Ma. In contrast, when the initial width is greater than 750 km, the corner flow produced by the western slab has little effect on the eastern slab. In this case, the dense eastern slab breaks off and sinks into the deep mantle in less than 40 Ma (fig. S16). Thus, the proximity of subduction zones in Myanmar likely permitted preservation and identification of the older subducting slab.

On the basis of our results, the discovery of two subducted slabs in close proximity beneath Myanmar is a unique feature preserved in the upper mantle beneath the tectonic domain of the eastern Himalayan syntaxis. Such a feature is not ubiquitously observed along the Himalayan collision belt because the collision and postcollision deformation tend to detach the previous subducted slabs. Geodynamic modeling further testified that two subducted slabs can be preserved under some special tectonic conditions. Our findings suggest the existence of two subduction zones during the India-Eurasia collision. Such an additional subduction system in the Neo-Tethyan Ocean has been invoked to explain the palaeomagnetically inferred rapid India-Eurasia convergence rates (*53*, *54*). The double subducted slabs in the upper mantle provide the compelling evidence for such a multistage subduction system.

## MATERIALS AND METHODS

### Finite-frequency tomography

In this research, we assemble seismic data from the newly deployed CMGSMO seismic network, the China Digital Seismic Network (CSN) (*55*), the ChinArray project (*56*), and the Incorporated Research Institutions for Seismology (IRIS) data management center (fig. S1). The CMGSMO arrays we used here comprise a dense linear subarray and two off-line subarrays (fig. S1 and table S2). Most of these stations have recorded ~18-month continuous raw seismic data. For CMGSMO I, the station spacings are 10 to 15 km for the linear subarray along a latitude of 22°N and 20 to 40 km for the off-line subarray in the Indo-Burman Range and Central Lowland. The station spacings of the off-line subarray of the CMGSMO II in the Shan Plateau are 50 to 70 km. Benefitting from the boost of seismic investigations in the surrounding regions, we also established a vast supplemental dataset including 58 permanent stations from the CSN (*55*), 342 portable stations from the ChinArray project (*56*), and 211 permanent or portable stations from the IRIS. As most of teleseismic events recorded by the CMGSMO arrays are from southeast, it is necessary to involve the CSN and ChinArray in the east and northeast of the CMGSMO arrays. The southwest incident data recorded by the CSN and ChinArray balance the data azimuthal coverage, thus reducing smearing along the dominant azimuth. The stations of IRIS are mainly distributed northwest of study area (fig. S1). Because the teleseismic events are mainly from southeast, the IRIS dataset will greatly help to image the deeper structure of the upper mantle beneath the Myanmar region. Considering that our dataset combines ~13 years of campaign data collected during different periods, we used the permanent station LSA from IRIS as a common reference when measuring relative travel time. From the total of 695 seismic stations, 45,141 high-frequency and 39,901 low-frequency *P*-wave arrivals were picked from 2660 earthquakes with epicentral distances of 30° to 90° and magnitudes greater than 5.5 (fig. S1). *P*-wave arrivals were measured using a cross-correlation method [Automated and Interactive Measurement of Body-wave Arrival Times (AIMBAT) software] (*57*) from vertical component recordings within frequency ranges of 0.1 to 0.5 Hz (low) and 0.5 to 2.0 Hz (high).

A seismic wave is composed of finite-frequency signals, and its travel time is affected by 3D volumes surrounding the ray path due to scattering when propagating through 3D Earth media. To model this realistic wave propagation, Dahlen *et al.* (*58*) and Hung *et al.* (*59*) adopted linear Born single-scattering theory and proposed a formulation that describes the relation between finite-frequency seismic wave travel-time shifts and velocity perturbations off the central geometric ray

$$\delta t={∭}_{⊗}K(x,\omega )\;\delta c(x)/c(x)d^{3}x$$

where *K*(**x**, ω) is the 3D Fréchet sensitivity kernel and represents the contribution of velocity anomalies at scattering point **x** to the travel-time shift.

For teleseismic finite-frequency tomography at the regional scale, the relative arrival times of body-wave phases between station pairs are used to invert for the subsurface mantle velocity structure. For the relative arrival time between two adjacent stations 1 and 2, δ*t*_1_ − δ*t_c_*, the sensitivity kernel can be simply expressed as the difference between the Fréchet kernels corresponding to each travel-time shift, δ*t*_1_ and δ*t*_2_ (*36*, *58*)

$$K_{\delta t_{1}-\delta t_{2}}=K_{\delta t_{1}}-K_{\delta t_{2}}$$

### Model parameterization and inversion

We parameterized the model space beneath our study area into a 3D regular grid (33 by 33 by 33) centered at 97.0°E, 27.5°N with spans of 19.2° in both latitude and longitude and with depth of 1920 km from the surface. The grid spacings were 66 km, 66 km, and 60 km in latitude, longitude and depth, respectively. The inversion problem with this parameterization can be written as

$$d_{i}=G_{\mathrm{il}}m_{l}$$

where *d_i_* represents the *i*th differential travel-time datum, and *m_l_* is the model parameter at the *l*th grid node. *G_il_* is the differential value of the integrated volumetric sensitivity contributing to the *l*th node (*36*).

During the inversion, the damped least square method (*60*) was used

$$\hat{m}={\left(G^{T}G+\theta^{2}I\right)}^{-1}G^{T}d$$

where **I** is the unit matrix and θ is the damping factor, which is determined by the trade-off analysis between the model *L*_2_ norm and travel-time variance reduction (fig. S17). The inverted results are discussed later with a damping factor that leads to a variance reduction of ~73%. According to the statistical results for travel times, the mean value and SD are −0.01833 and 0.67240 before the inversion and −0.01824 and 0.51429 after the inversion, respectively (fig. S18).

### Crustal and elevation corrections

The travel-time anomalies recorded by the stations may come from station elevations, heterogeneous crustal structures, and lateral inhomogeneities of the upper mantle structure. Although teleseismic tomography resolves upper mantle structure well, its imaging ability for the crust and uppermost mantle is barely satisfactory due to poorly crossing teleseismic rays at shallow depths. To minimize the impact of the shallow velocity structure on the upper mantle velocity structure, we corrected the travel-time residuals by the frequency-dependent crustal correction method (*61*) and the crust tomographic model (*41*). We used CRUST1.0 (*62*) for the stations where a crustal velocity model is not defined. In addition, station terms were included in the inversion to imbibe travel-time shifts caused by shallow heterogeneous structures.

### Resolution tests

The data coverage and the reliability of the imaged results were estimated by performing 3D resolution tests (fig. S3). Synthetic travel times were computed for different input velocity models with the same Fréchet kernels and source/receiver pairs as those used to invert the actual dataset

$$\Delta t_{\text{syn}}=G\cdot \Delta c_{\text{syn}}+t_{\text{noise}}$$

where **t**_noise_ is a Gaussian random noise and the SD of **t**_noise_ for the *P* wave is 0.1 s, which is approximately 10% of the typical relative travel-time delay span for an event (*10*). The inversion was then implemented for the synthetic dataset using the same inversion parameters (smoothing and damping parameters) as those used for the real dataset.

Checkerboard resolution tests were executed with checkers of two different sizes to estimate the robustness of large- and small-scale features in the tomographic results (fig. S3). One test had a lateral grid spacing of ~130 km by 130 km and a thickness of ~120 km. Another test had lateral sizes of ~200 km by 200 km and a vertical thickness of ~120 km. Both checkers had input velocity perturbations of ±3% for the *P* wave, with the magnitude decreasing from the center of each checker to zero at its boundary as a cosine function. The checkerboard results illustrate that large-scale anomalies are well retrieved in all profiles, while smaller anomalies can be well recovered above a depth of 400 km.

### Reliability of tomographic results

Tomographic studies should offer objective evidence for the reliability of the velocity models. The similarities among various velocity models produced by different groups and datasets for the same area can be forceful indicators to judge the reliability of tomographic models. Our tomography models clearly identify HV1 beneath the Indo-Burman Range. Previous global and regional tomographic results (*13*, *15*) also imaged such a high-velocity structure, although discrepancies exist in dip angle, thickness, depth of termination, and velocity perturbation (fig. S4). HV1 inverted by previous studies generally has lower velocity perturbations of 1 to 2% or even lower than ~0.5%, a wide slab thickness of ~200 km, and an ambiguous dip angle (fig. S4). We note that our model shows a more compact HV1 with higher amplitude than those of other models and clearly reveals the variations in the dip angle of the subducted slab and its depth of termination.

We further evaluated the robustness of the results by synthetic tests. Synthetic travel times were generated for different input velocity models with the same Fréchet kernels and source/receiver pairs as those used to invert the actual dataset. Then, the same inversion method used for the observed dataset was implemented for the synthetic dataset. On the basis of the characteristic anomalies revealed in four profiles AA′, BB′, CC′, and DD′ (Fig. 2, D to G), we conducted synthetic tests with the input anomalies of the same parallel east-dipping anomalous velocity bodies (two high-velocity bodies) in the upper mantle (fig. S5) to assess our ability to determine the structure of the two notable anomalies in our tomographic models. More recovery experiments with custom models were presented (figs. S6 to S9) to further evaluate how well the dip angles and depth of the featured structures beneath Myanmar are constrained.

We first tested the degree of eastward-directed smearing for the imaged velocity anomalies by assuming a discontinuous eastern high-velocity anomaly (fig. S6). The recovery test reveals that eastward-directed smearing for HV2 above depths of ~300 km is negligible except in profile AA′. Thus, continuous extension of HV2 to ~300 km, as inferred in our study, is reliable. We then tested different dip directions for the featured structures. The input models consisted of west-dipping anomalies (fig. S7) and a model of two parallel vertical high-velocity layers (fig. S8). The dip directions of the recovered models were indistinguishable from the input models, so dip angles are also robust in the observed models. In addition, we tested whether HV2 might be an artifact arising from interactions between two parallel eastward-dipping high-velocity (+2%) and low-velocity (−2%) bodies (fig. S9). This test shows that HV2 is not an artifact induced by a low-velocity structure. These experiments indicate that the specified east-dipping structures are reliable in our study.

### Waveform modeling

We chose two teleseismic events, 2017/08/16 and 2017/09/07, with similar azimuthal angles of approximately 270°, different epicentral distances, and similar ray paths in the receiver-side upper mantle roughly parallel to the two east-dipping high-velocity anomalies (HV1 and HV2). Event 2017/08/16 occurred 158 km WNW of Naze, Japan, on 16 August 2017 [2017-08-16, 12:51:25.87, 28.674°, 127.901°, 198.0 km, moment magnitude (*M*_w_) of 5.7], and event 2017/09/07 was located 250 km WNW of Chichi-shima, Japan and occurred on 7 September 2017 (2017-09-07, 17:26:49.32, 27.783°, 139.804°, 451.0 km, 6.1 *M*_w_) (fig. S10). The raw waveforms recorded at stations M00 to M44 were deconvolved with their instrumental responses and bandpass-filtered between 0.008 and 0.8 Hz using a Butterworth filter. *P* waveform distortions varying with epicentral distance are observed along the east-west–trending linear subarray of the CMGSMO (M00 to M44; 38 stations). The distinct epicentral differences of the selected events negate the contributions from source-side structures to the observed waveform distortions (fig. S10B). No similar waveform distortions can be detected from the earthquakes located west of stations, ruling out contributions from receiver-side crustal heterogeneities. These observations place tight constraints on the seismic characteristics of HV1 and HV2 from the tomographic results.

We used a finite difference method with a grid size of 1 km and a time step size of 0.01 s to conduct 2D forward modeling of *P* waveform data (*39*). We first calculate the contributions of crustal structures to anomalous *P* waveforms using a 2D model combining a crustal velocity model from local tomography (0 to 35 km) (*41*) with a 1D upper mantle model (*63*). The synthetics deviate strongly from observations, confirming the minor contributions of crustal structures (fig. S10, D and F). After we updated the 2D model by replacing the 1D upper mantle model by our slightly modified tomographic results, the synthetics match the observations well (fig. S10, E and G). In addition, we built several models based on upper mantle *P*-wave images of previous studies (*13*, *15*). Waveform modeling results by these models also deviate strongly from the characteristic phases (fig. S19, A and B).

We further conducted waveform modeling based on a series of 2D synthetic models to quantify the acceptable ranges of thickness, velocity perturbation, and penetration depths of HV1 and HV2. For HV1, the model thickness varied from 10 to 150 km with an increment of 20 km, the velocity perturbation varied from 0 to 6% with an increment of 0.5%, and the penetration depth varied from 360 to 600 km with an increment of 20 km. For HV2, the thickness varied from 0 to 120 km with an increment of 20 km, the velocity perturbation varied from 0 to 3.5% with an increment of 0.25%, and the extending depth varied from 200 to 500 km with an increment of 20 km. Velocity perturbations of HV1 and HV2 satisfy Gaussian distribution at the same depth. We defined objective functions to determine the fitting between the observations *O*(*t*) and synthetics *S*(*t*). The objective function is defined as

$$\text{OBJ}=\frac{m\sum O(t)\cdot S(t)}{\sum O^{2}(t)+\sum S^{2}(t)}$$

where *m* is the weight chosen based on the observations.

We set the acceptable threshold to be the objective functions higher than 0.9 for HV1 and 0.88 for HV2. The optimal velocity perturbation, extending depth, and thickness for HV1 are 3.6 to 5.1%, 360 to 480 km, and 80 to 150 km, respectively. The optimal values for HV2 are 1.2 to 2.7%, 230 to 390 km, and 60 to 120 km, respectively (fig. S13).

In addition, we conducted a synthetic test to determine the influence of waveguide effect on waveforms. We replaced the low-velocity structure between HV1 and HV2 with a high-velocity one to eliminate the waveguide structure (a low-velocity structure embedded with HV1 and HV2) (fig. S11). The sandwiched high-velocity structure has 2% velocity perturbation, same as that of HV2. The results show that the synthetic waveforms at stations M17 and M18 become broader and lower in amplitude compared to the observed waveforms (fig. S11). Thus, the observed narrow and high-amplitude waveforms at stations M17 and M18 could be related to waveguide effect.

### Estimation of the slab stalling time

Temperature is a key factor that controls upper mantle velocity perturbations (*46*). Because of mantle assimilation, a smaller velocity perturbation for a slab indicates residence of the slab in the upper mantle for a longer time. We conducted quantitative conversion between the velocity perturbation and thermal structure and slab stalling time. The width of the slab core was determined by the 2.5% contour for HV2 (Fig. 2G) and is ~60 km. We assumed that the oceanic slab has a thickness of 70, 80, or 90 km; calculated the initial thermal structure in the oceanic slab with a half-space cooling model; and then let the slab evolve conductively in the upper mantle for ~120 Ma (*63*). The core temperatures inside the slab remained 102°, 130°, and 167°C cooler than the surrounding mantle and implied maximum velocity perturbations of 0.5, 0.6, and 0.8% for the slab with thicknesses of 70, 80, and 90 km, respectively (*64*).

### Numerical modeling method

We used the thermomechanical code I2VIS (*65*) to model the dynamics of the double subduction system. The governing equations of conservation of momentum, mass, and energy were solved by the combination of a conservative finite difference method and a marker-in-cell technique applied on a staggered nonuniform Eulerian grid. I2VIS simulates creeping flow due to thermal and chemical buoyancy forces and takes into account the effects of adiabatic, shear, latent, and radioactive heating. The model domain was 5000 km wide and 1500 km deep and was resolved with a nonuniform 1251 by 481 rectangular grid. In the horizontal direction, the resolution was 4 km. In the vertical direction, the resolution was 1 km for the upper 200 km, decreasing to 5 km between a depth of 200 and 300 km, and 5 km over the remaining grid. More than 10 million Lagrangian markers were randomly distributed in the whole model domain and were used to advect material properties defined at the Eulerian mesh. The deformation of all materials was governed by viscoplastic rheology, which accounts for plastic yielding at shallow depths and low temperatures, and dislocation creep at greater depths and temperatures. All the details of the method, allowing for the reproduction, can be found in the study of Gerya (*66*).

### Model configuration, initial conditions, and boundary conditions

The initial model consisted of two oceanic slabs and three continental terranes. The western oceanic slab was ~530 km wide, and the eastern oceanic was ~130 km wide. Both the western and eastern slabs had a 7-km-thick oceanic crust and 83-km-thick lithospheric mantle and were prescribed to have subducted to a depth of ~200 km. To enable the double subduction, the eastern slab was set to be 50 kg/m^3^ denser than the western slab. All the continental terranes were composed of a 40-km-thick crust (the upper and lower crusts were 20-km thick each) and a 100-km-thick lithospheric mantle, giving a total lithospheric thickness of 140 km. The width of the middle continental terrane was variable in different tests. The initial temperature increased linearly from 0°C at the model surface to 1350°C at the lithosphere base. An adiabatic gradient of 0.5°C/km was used for the sublithosphere mantle. All mechanical boundary conditions were free slip. The thermal boundary conditions were the following: constant temperature (273 K) on the top, remote fixed temperature on the bottom (*67*), and insulating (no horizontal heat flow) on both sides. The surface of the rocky portion was treated as an internal free surface by placing an overlying 20-km-thick “sticky air” layer (*68*), which is characterized by low viscosity (10^18^ Pa ∙ s) and a low density of 1 kg ∙ m^−g^. A constant convergence rate of 3.5 cm/year was imposed at the western side of the model to drive the double subduction system (fig. S14).

## Supplementary Materials

### This PDF file includes:

Figs. S1 to S19 Tables S1 and S2 References
