# Supplementary material for: Slab remnants beneath the Myanmar terrane evidencing double subduction of the Neo-Tethyan Ocean
Source: Sci Adv. 2022 Aug 26;8(34):eabo1027. doi: 10.1126/sciadv.abo1027 (PMC9417170; doi:10.1126/sciadv.abo1027)
Supplement: Supplementary file 1 — Figs. S1 to S19 Tables S1 and S2 References [file sciadv.abo1027_sm.pdf]

Science Advances
AAAS

Supplementary Materials for

Slab remnants beneath the Myanmar terrane evidencing double subduction of the Neo-Tethyan Ocean

Shun Yang *et al.*

Corresponding author: Mingming Jiang, jiangmm@mail.iggcas.ac.cn; Yumei He, ymhe@mail.iggcas.ac.cn

Sci. Adv. **8**, eabo1027 (2022)
DOI: 10.1126/sciadv.abo1027

This PDF file includes:

Figs. S1 to S19
Tables S1 and S2
References

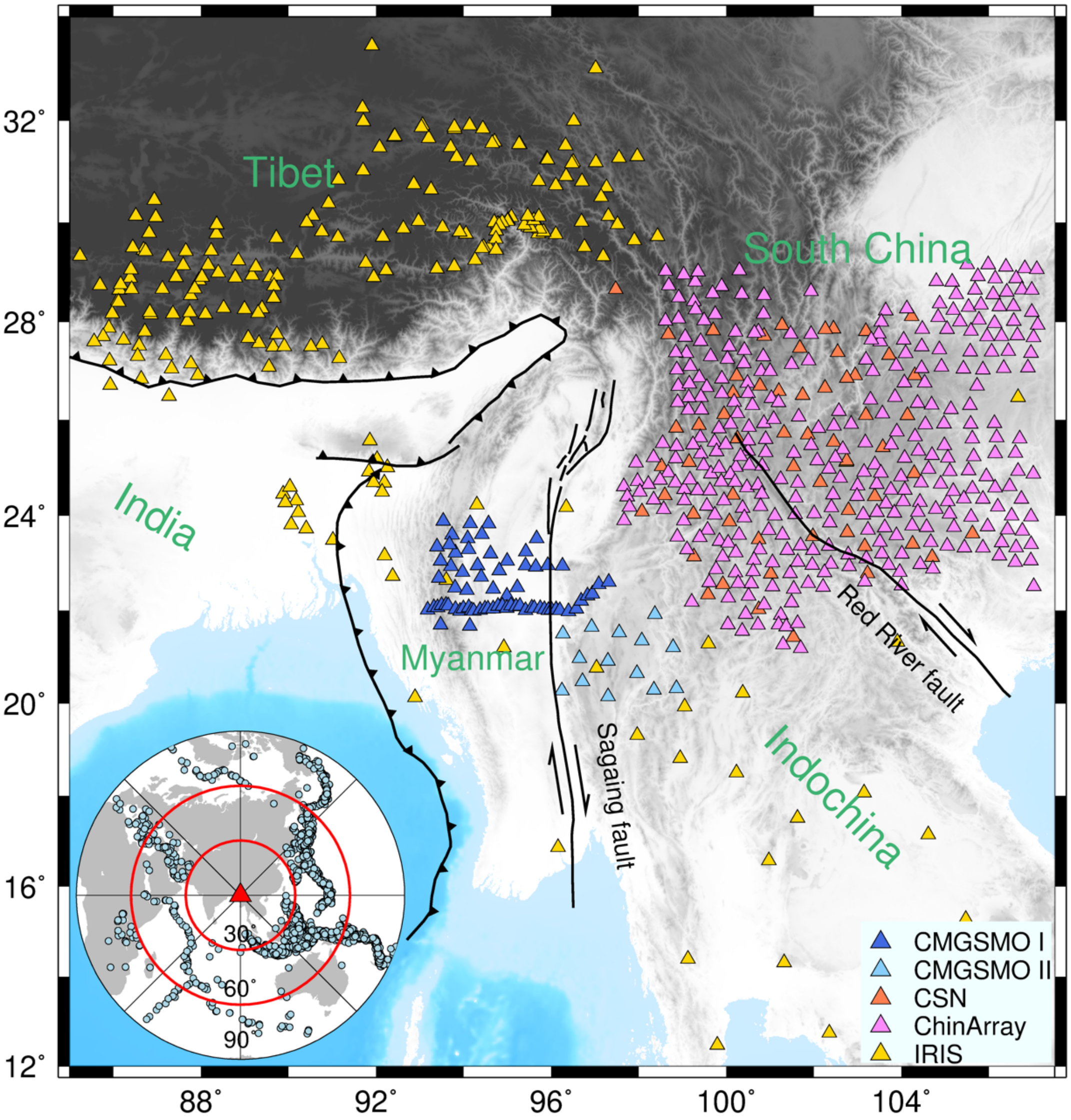

Fig. S1. Distribution of seismic stations (triangles) and events (light blue dots) used in this study.

Triangles with different colors show seismic stations from different projects. The bottom-left inset represents collected teleseismic events with epicentral distances of 30° – 90° and magnitudes greater than 5.5.

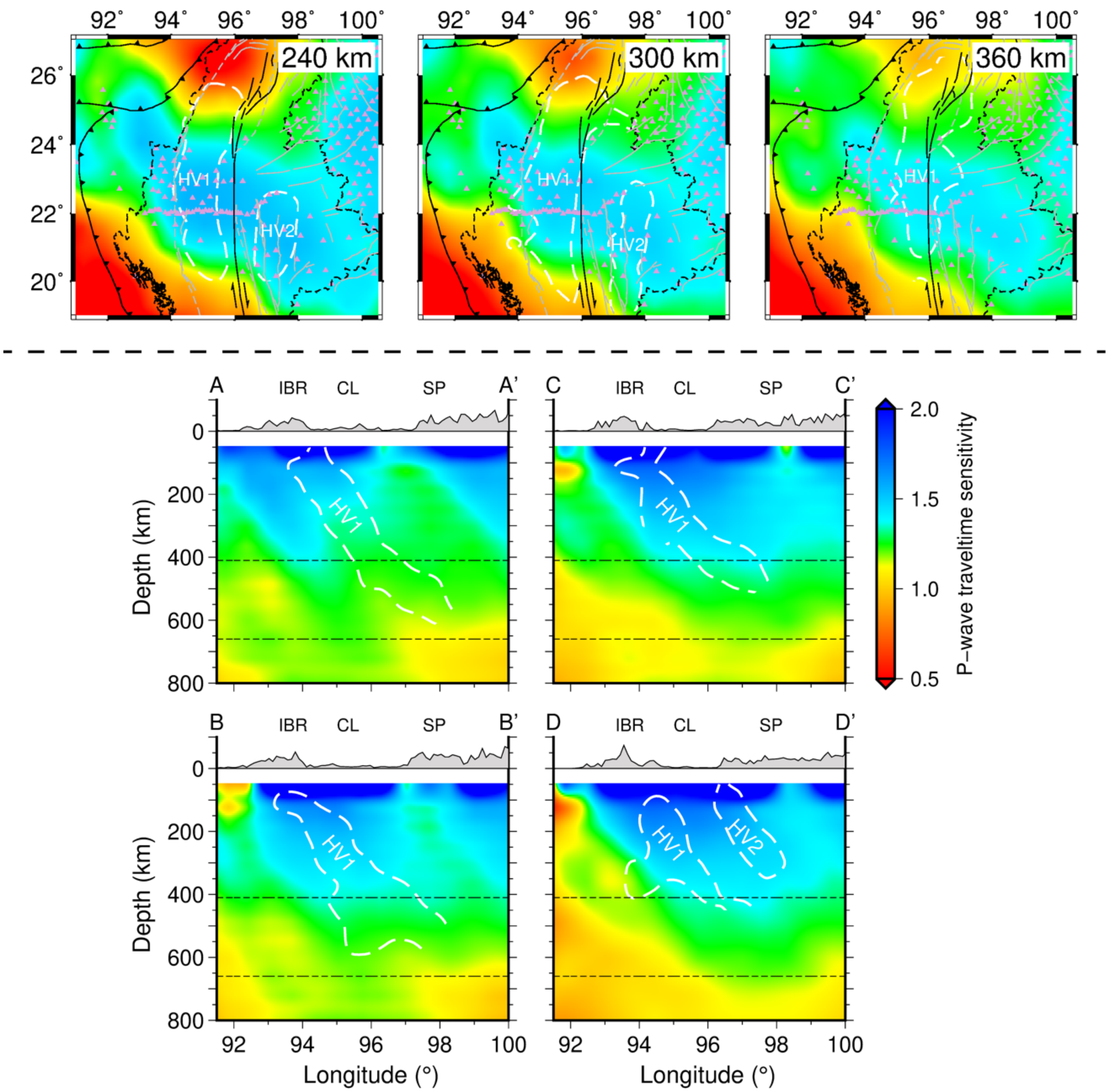

Fig. S2. The distribution of P-wave traveltime sensitivity.

Top panel: The distribution of P-wave traveltime sensitivity in horizontal slices at different depths. The gray and black solid lines represent geological structures. The light-purple triangles represent the distribution of seismic stations. Bottom panel: The distribution of P-wave traveltime sensitivity in vertical slices. The results show strong traveltime sensitivity in the central and eastern parts of the velocity model, reflecting the robustness of models in the areas occupied by HV1 and HV2. Locations of AA', BB', CC' and DD' are shown in Fig. 2C. The white dashed lines mark 1% contours of HV1 and HV2. IBR: Indo-Burman Range; CL: Central Lowland; SP: Shan Plateau.

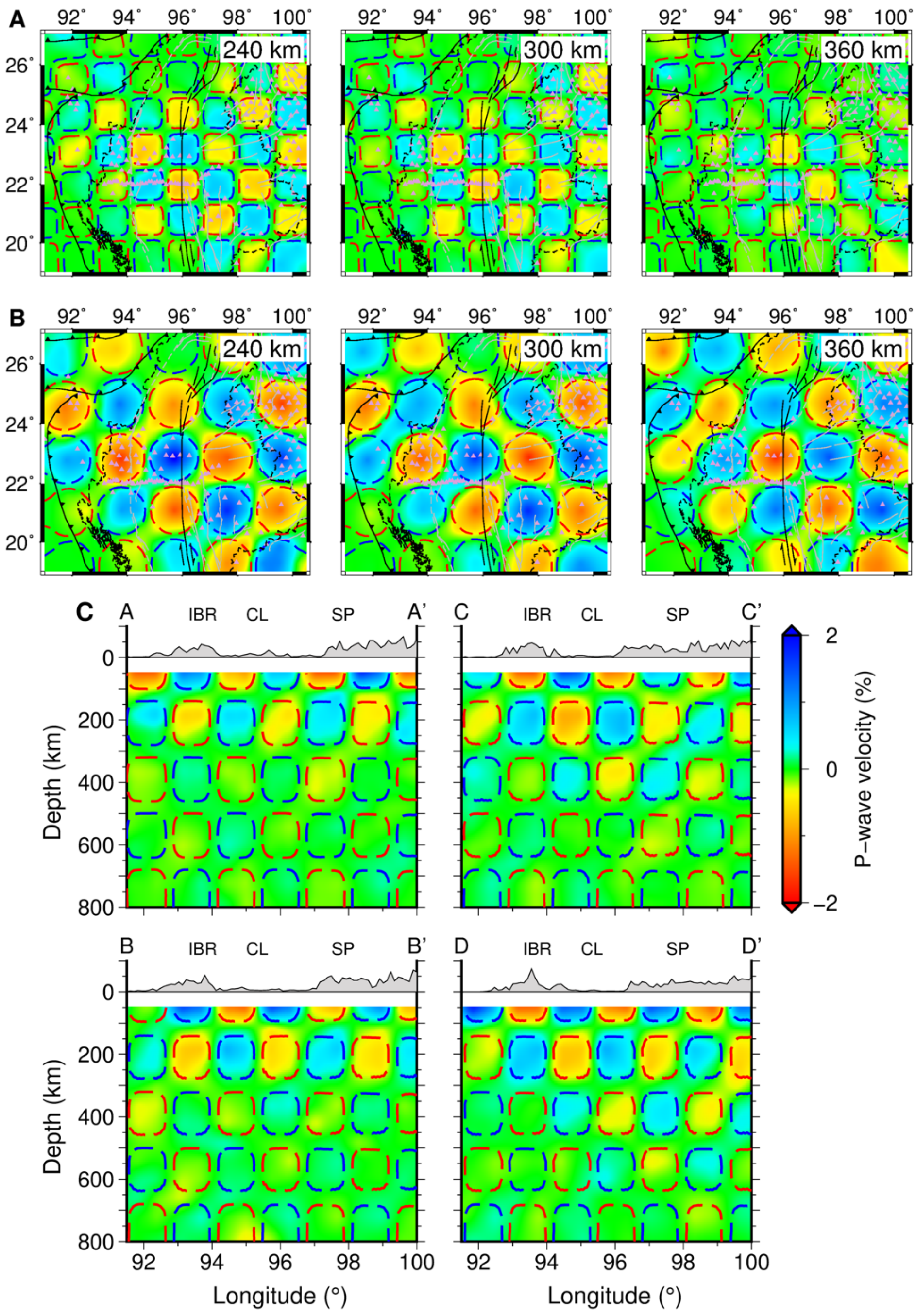

Fig. S3. Checkerboard tests.

(**A** and **C**) Inversion results of checkers with lateral sizes of ~130 km × 130 km and a vertical thickness of ~120 km in horizontal (**A**) or vertical (**C**) slices. (**B** and **D**) Inversion results of checkers with a lateral grid spacing of ~200 km × 200 km and a vertical thickness of ~120 km in horizontal (**B**) or vertical (**D**) slices. Both have input velocity perturbations of ±3% for the P-wave,

with the magnitude decreasing from the center of each checker to zero at its boundary as a cosine function. Blue and red dashed contour lines in each plot mark the original dimension of input checkers. The gray and black solid lines in (**A**) and (**B**) represent geological structures. The light-purple triangles in (**A**) and (**B**) represent the distribution of seismic stations. The location of profiles in (**C**) and (**D**) is illustrated in Fig. 2C.

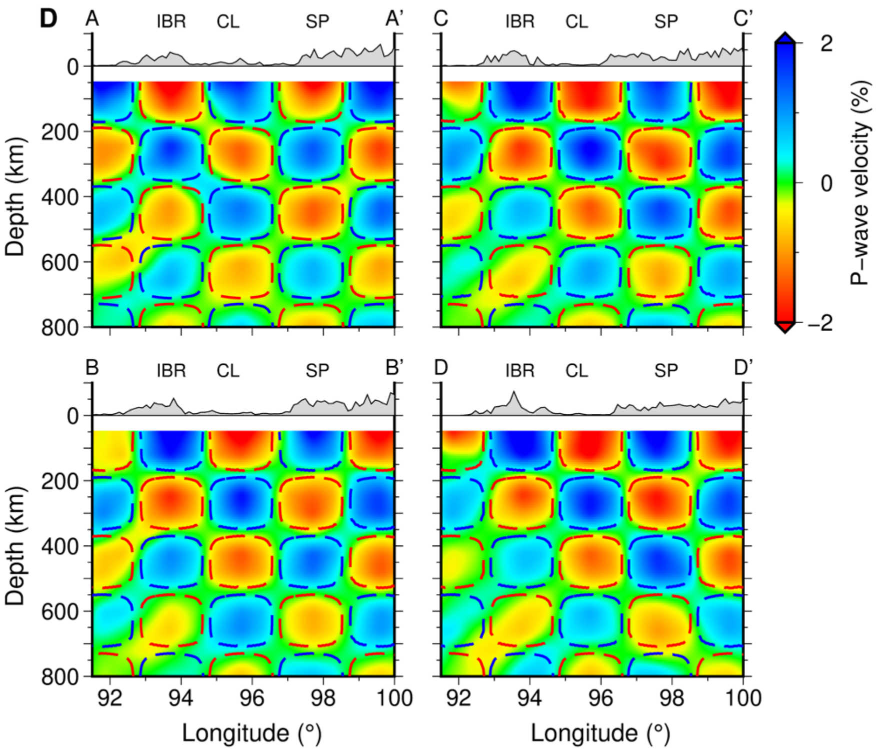

Fig. S3. (*continued*)

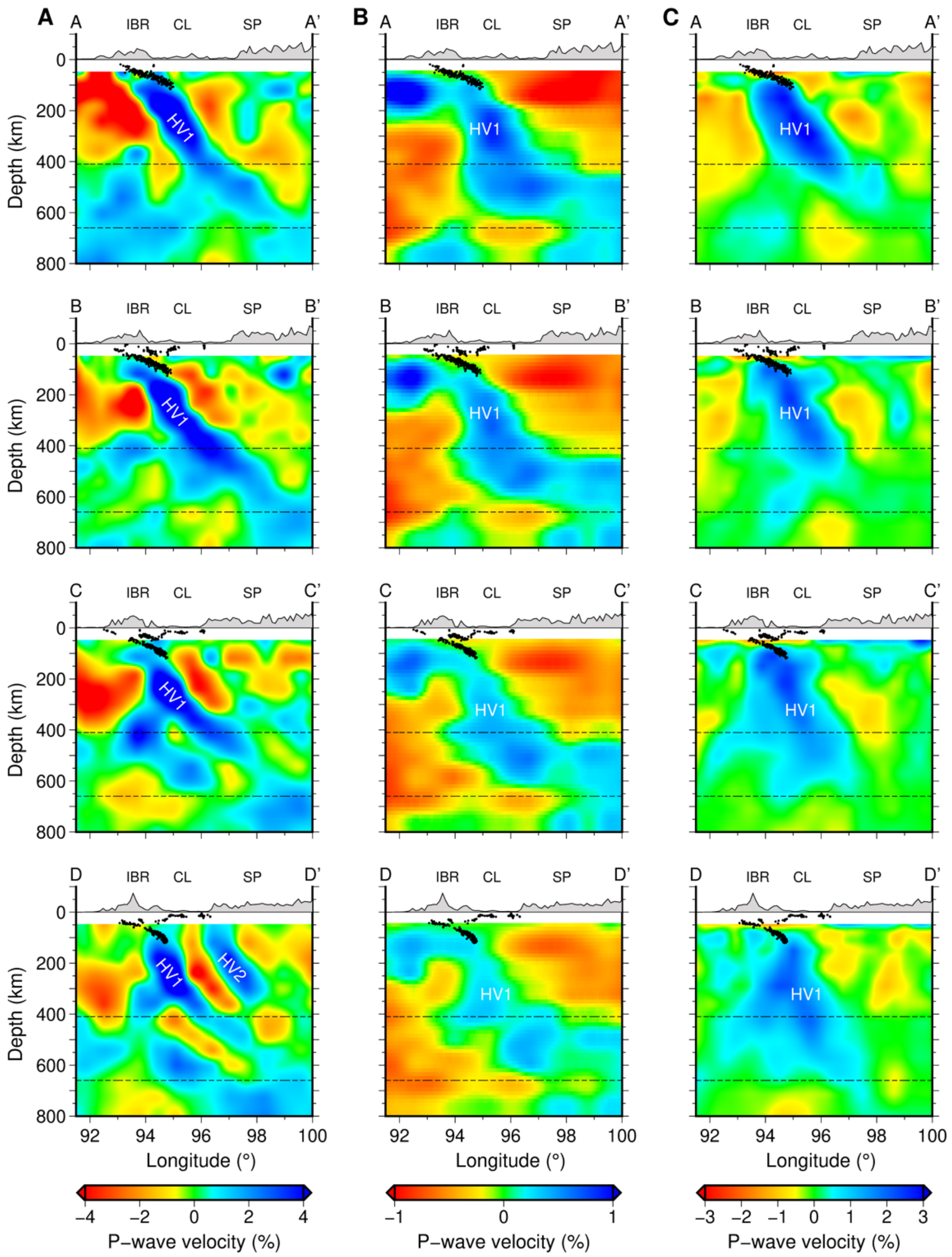

Fig. S4. Comparison between the P-wave velocity models obtained here and previous studies along the same cross-sections.

(**A**) P-wave velocity models from our study. (**B** and **C**) P-wave velocity models of Li et al. (*13*) and Koulakov (*15*). Locations of AA', BB', CC' and DD' are shown in Fig. 2C. Black dots mark the locations of earthquakes (*35*).

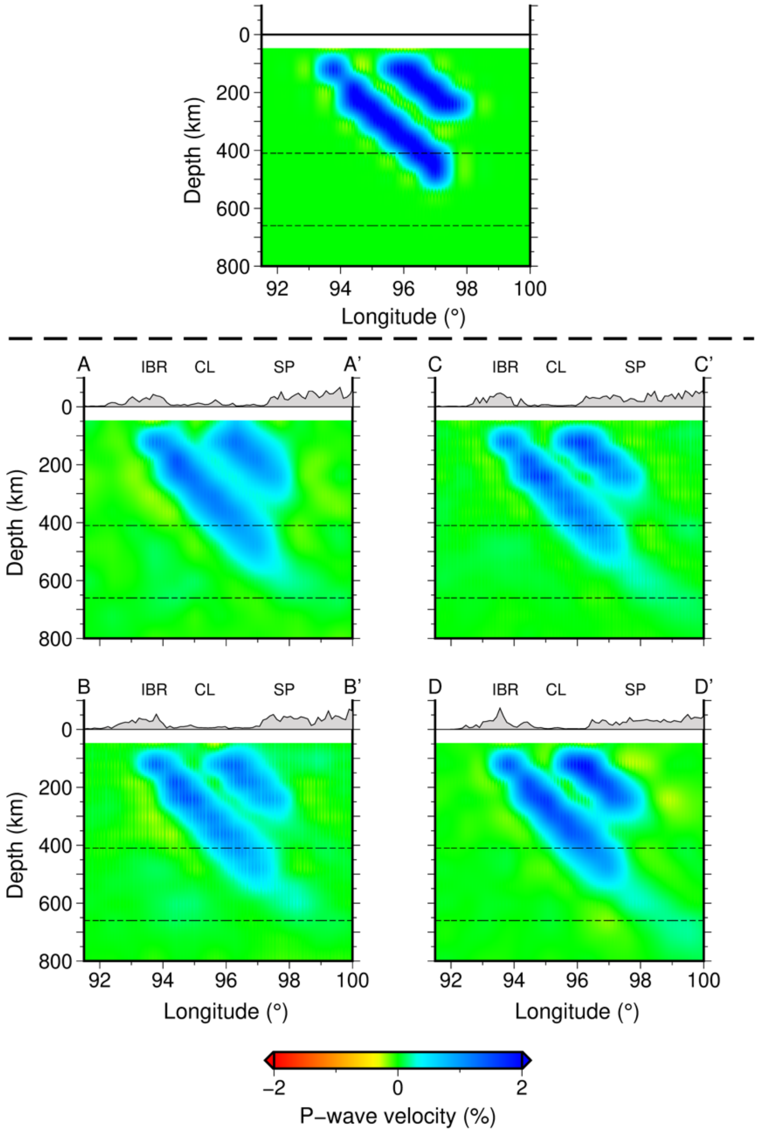

Fig. S5. A synthetic experiment testing the recoverability of the morphology of two distinct P-wave velocity anomalies in final tomographic images.

Top panel: An input model with two parallel east-dipping velocity bodies with similar geometry as the inverted model shown in Figs. 2D-G. The input velocity perturbations are 2% for high-velocity structures. Bottom panels: Inverted models showing good recovery of the morphology of the featured structures. Here the location of profiles AA', BB', CC' and DD' are shown in Fig. 2C. Black dashed lines depict the 410 km and 660 km discontinuities.

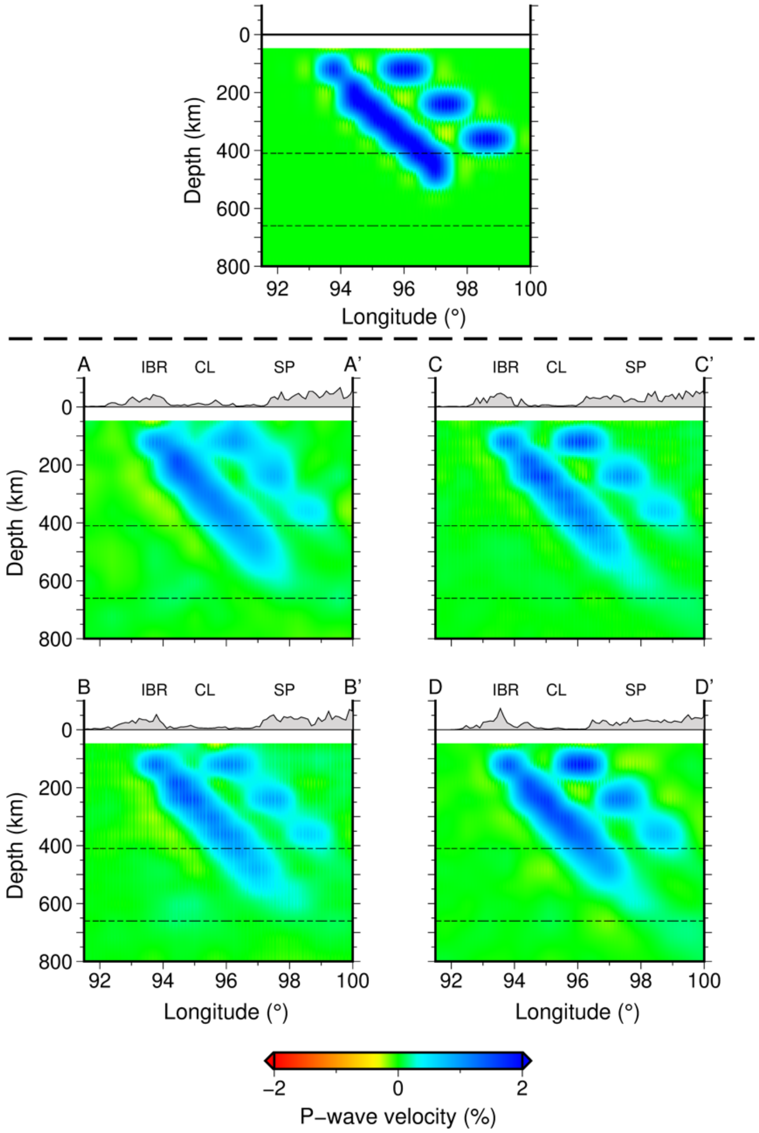

Fig. S6. A synthetic experiment testing the recoverability of a discontinuous HV2 anomaly. Top panel: An input model with two east-dipping velocity bodies, but with a discontinuous eastern high-velocity anomaly. The input velocity perturbations are 2% for high-velocity structures. Bottom panels: Inverted models showing HV2 has notable smearing below ~300 km. The location of profiles is illustrated in Fig. 2C.

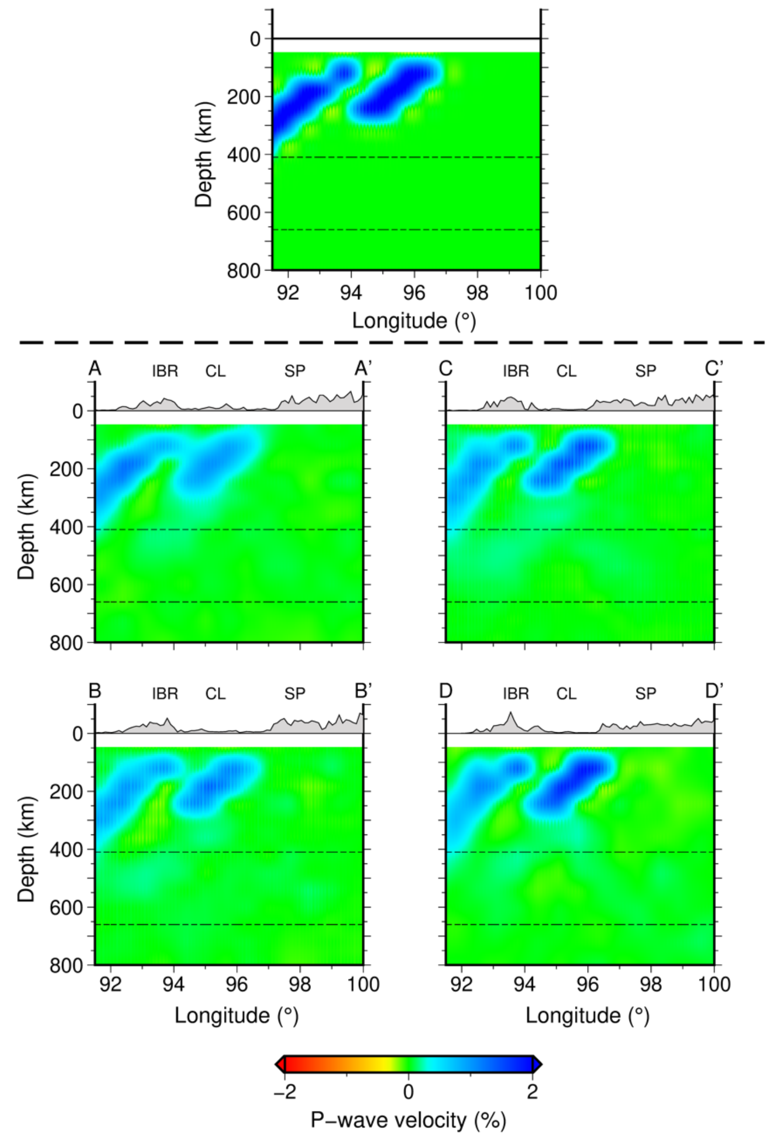

Fig. S7. A synthetic experiment testing the recoverability of dip directions of west-dipping high P-wave velocity anomalies.

Top panel: An input model with two west-dipping velocity bodies. The input velocity perturbations are 2% for high-velocity structures. Bottom panels: Inverted models showing that dip directions are invariant compared to the input models. The location of profiles is illustrated in Fig. 2C.

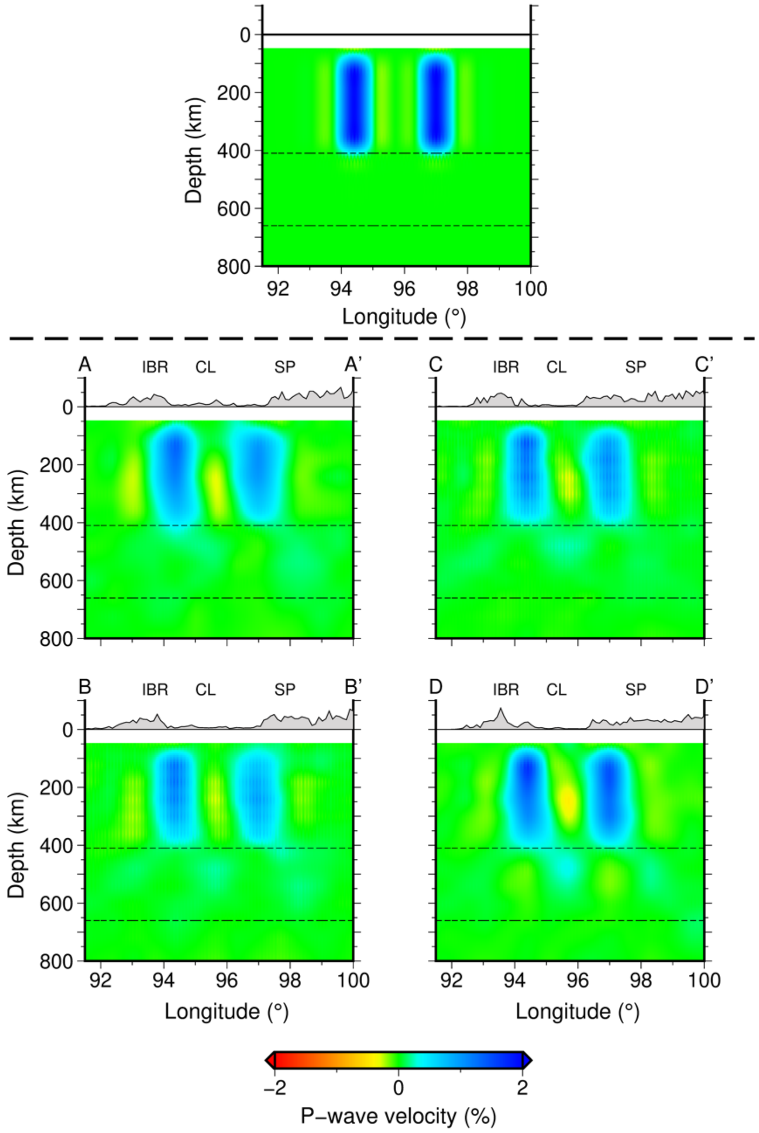

Fig. S8. A synthetic experiment testing the recoverability of dip directions of vertical high P-wave velocity anomalies.

Top panel: An input model with two vertical velocity bodies. The input velocity perturbations are 2% for high-velocity structures. Bottom panels: Inverted models showing that dip directions are invariant compared to the input models. The location of profiles is illustrated in Fig. 2C.

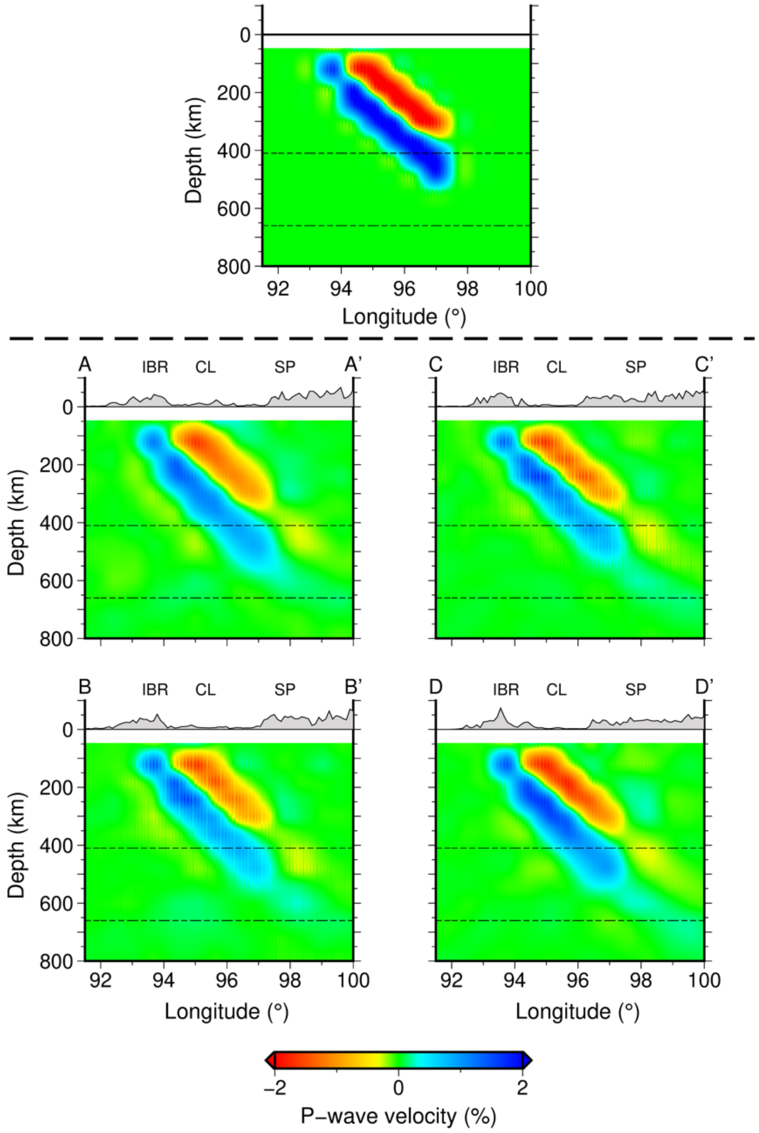

Fig. S9. A synthetic experiment testing whether HV2 is an artifact due to the emergence of a low-velocity structure.

Top panel: An input model with two parallel eastward dipping high-velocity and low-velocity bodies. The input velocity perturbations are 2% and -2% for high-velocity and low-velocity structures, respectively. Bottom panels: Inverted models showing the existence of low-velocity anomaly does not induce the appearance of HV2. The location of profiles is illustrated in Fig. 2C.

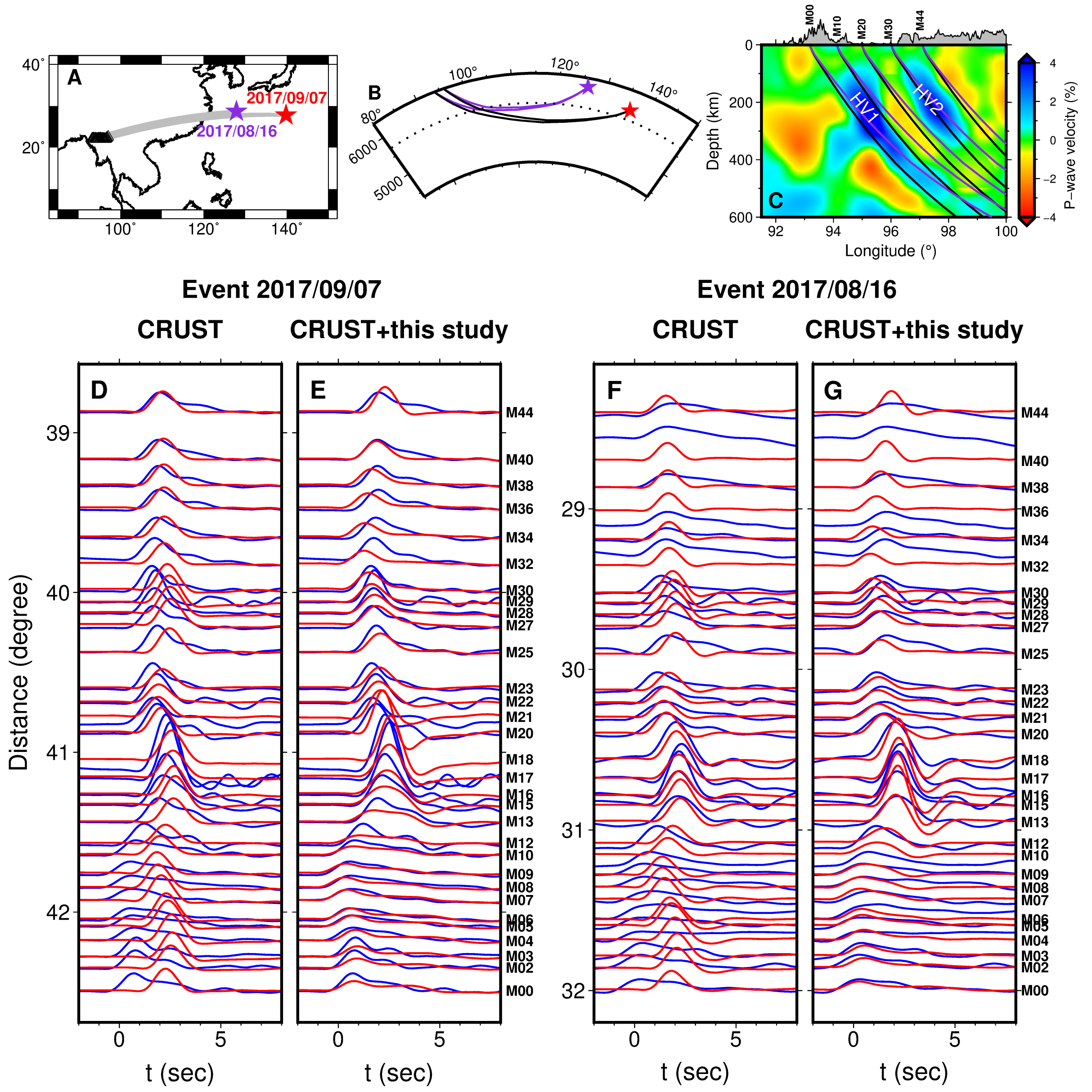

Fig. S10. Two-dimensional waveform modeling of events 2017/08/16 and 2017/09/07 along the high-density seismic array M00-M44.

(**A**) The selected events 2017/08/16 and 2017/09/07 used in this study (red and purple stars), seismic stations (black triangles) and great circle paths (gray lines). (**B**) Ray paths of direct P at epicentral distances from 30° to 32° for event 2017/08/16 and 40° to 42° for event 2017/09/07. These ray paths are calculated based on the IASP91 model (*73*). (**C**) The tomographic model along

the profile at 22ºN, and the low-velocity perturbation between HV1 and HV2 is slightly weakened. (**D** and **F**) Comparison between observed (blue lines) and synthetic (red lines) waveforms using only the crust velocity model from local tomography (*41*) and a 1-D upper-mantle velocity model (*63*). (**E** and **G**) Comparison between observed (blue lines) and synthetic (red lines) waveforms using a combined crust velocity model and upper mantle model shown in (**C**).

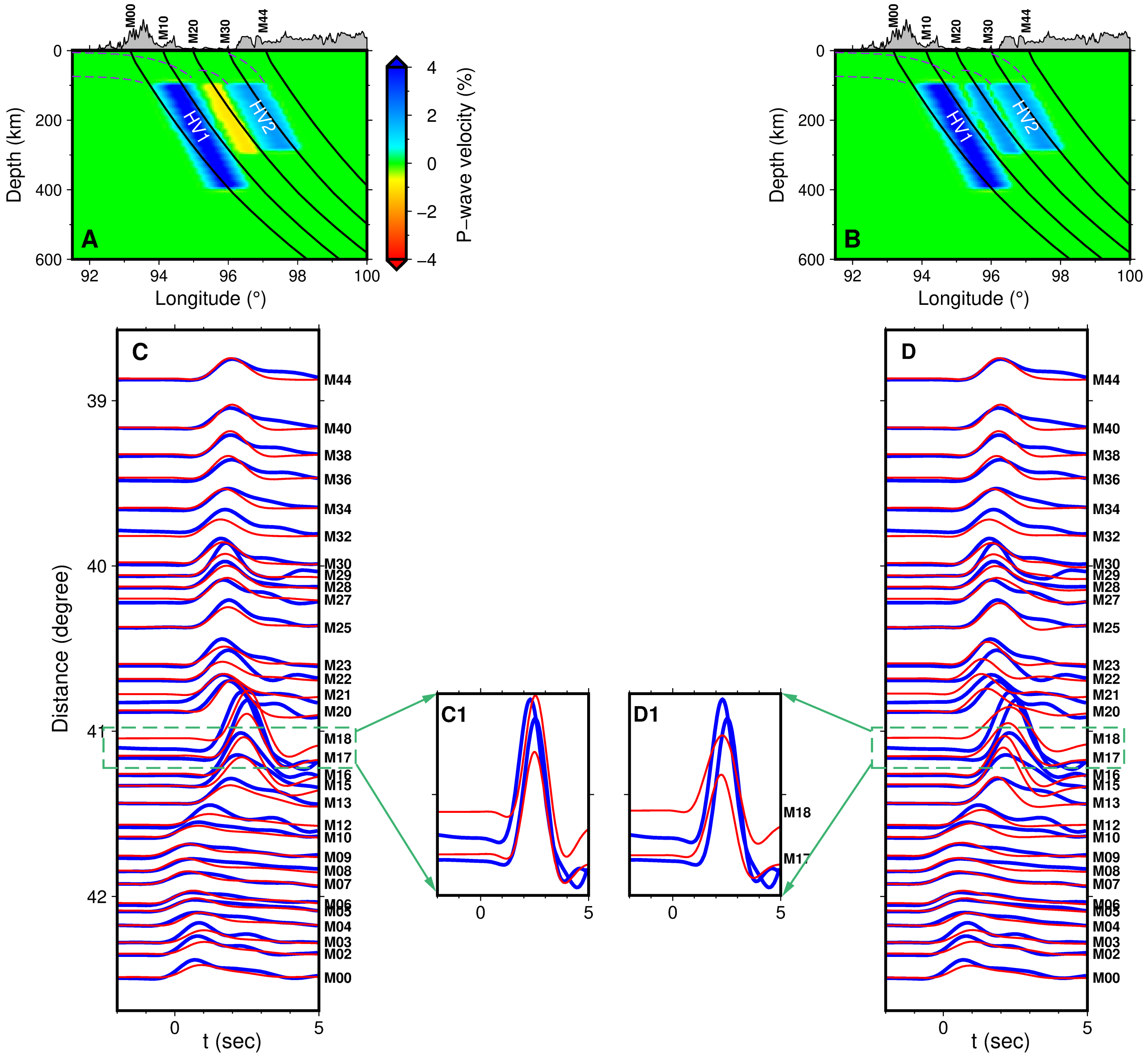

Fig. S11. Two-dimensional waveform modeling of event 2017/09/07 to test waveguide effect. The velocity model consisting of a low-velocity structure (**A**) or a high-velocity structure (**B**) sandwiched between HV1 and HV2 and the ray paths of direct P-waves at the receiver-side. The possible extensions of two slabs (HV1 and HV2) to a shallow depth are marked by dashed purple lines. The synthetic HV1 has a velocity perturbation of 4.5%, a thickness of 130 km and extends to 400 km depth, and HV2 has a velocity perturbation of 2.0%, a thickness of 120 km and reaches 300 km depth. (**C**) Vertical component comparison between the observation (blue lines) and

synthetics (red lines) based on the model combining the crustal model (*41*) and the upper mantle model shown in (**A**). (**D**) Vertical component comparison for the upper mantle model shown in (**B**). Detail views of waveforms at Station M17-M18 are shown in (**C1**) and (**D1**).

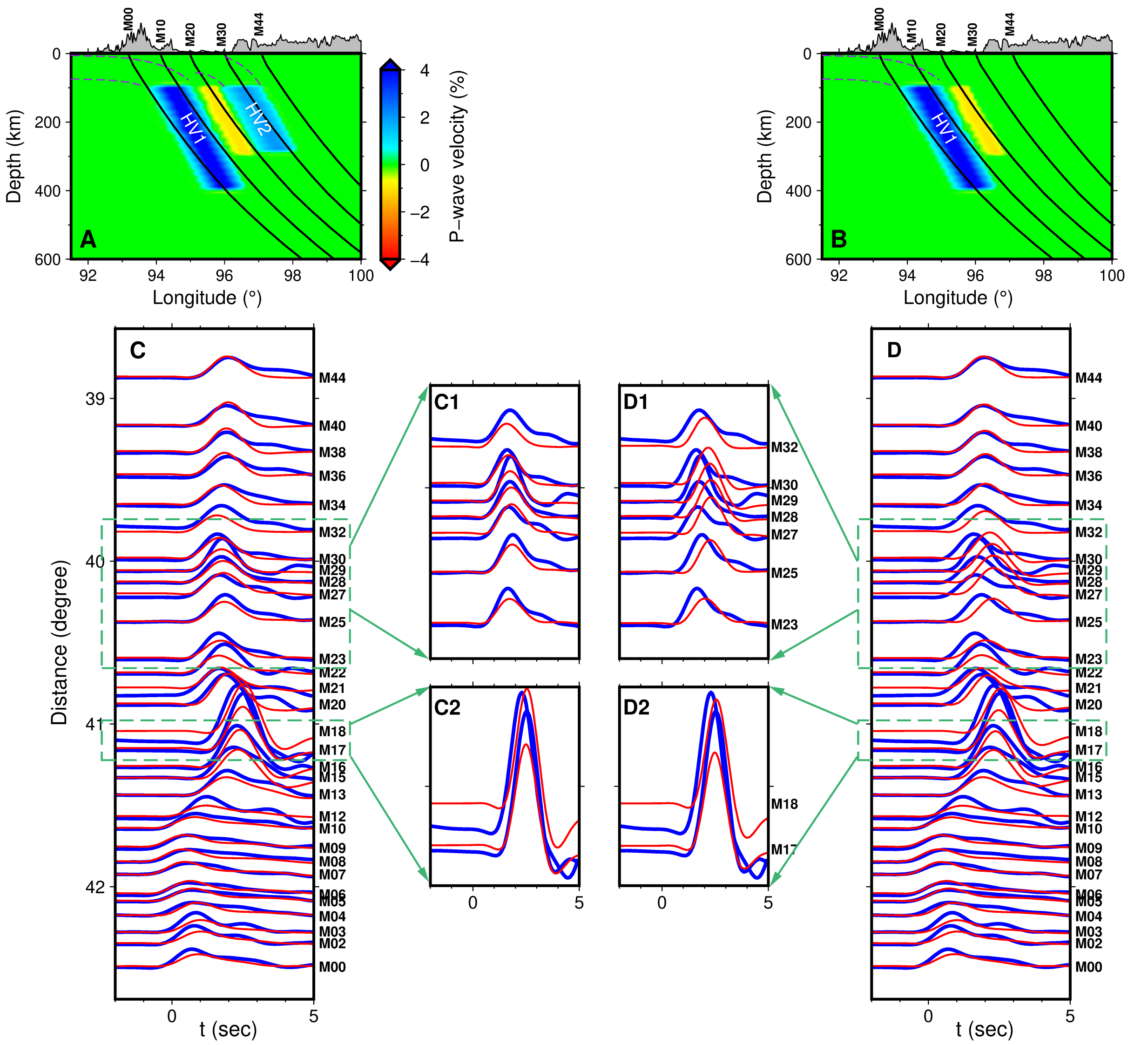

Fig. S12. Two-dimensional waveform modeling of event 2017/09/07 to test the influences of HV2 on waveforms.

The velocity model with HV2 (**A**) or without HV2 (**B**) and the ray paths of P-waves at the receiver-side. The possible extensions of two slabs (HV1 and HV2) to a shallow depth are marked by dashed purple lines. The synthetic HV1 has a velocity perturbation of 4.5%, a thickness of 130 km and extends to 400 km depth, and HV2 has a velocity perturbation of 2.0%, a thickness of 120 km and reaches 300 km depth. (**C**, **C1** and **C2**) Comparison between observed (blue lines) and synthetic (red lines) vertical displacements based on a model that combines the crust structure (*41*) and the

upper mantle model shown in (**A**). (**D**, **D1** and **D2**) Comparison between observed (blue lines) and synthetic (red lines) vertical displacements generated by a model that combines the crust structure and the upper mantle model shown in (**B**).

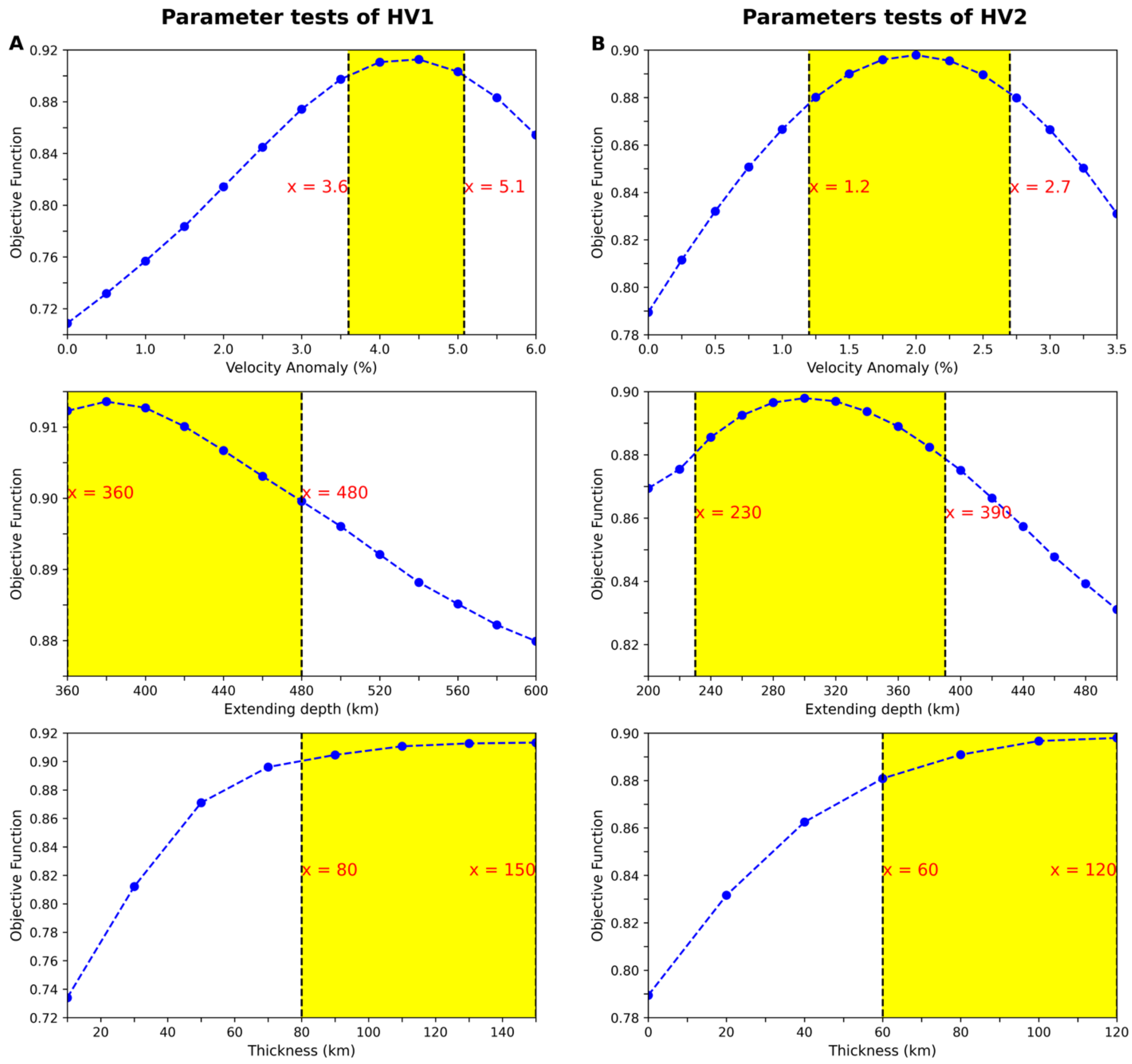

Fig. S13. Objective functions for synthetic 2-D velocity models with Gaussian-shaped velocity anomaly of different velocity perturbations, penetration depths, and thicknesses.

(**A** and **B**) Objective functions for HV1(**A**) and HV2 (**B**), respectively. The yellow zone marks the optimal values with objective functions greater than 0.9 (**A**) or 0.88 (**B**). HV1 has optimal velocity perturbations of 3.6-5.1%, thicknesses of 80-150 km, and penetration depths of 360-480 km. The corresponding parameters of HV2 are 1.2-2.7%, 60-120 km, and 230-390 km, respectively.

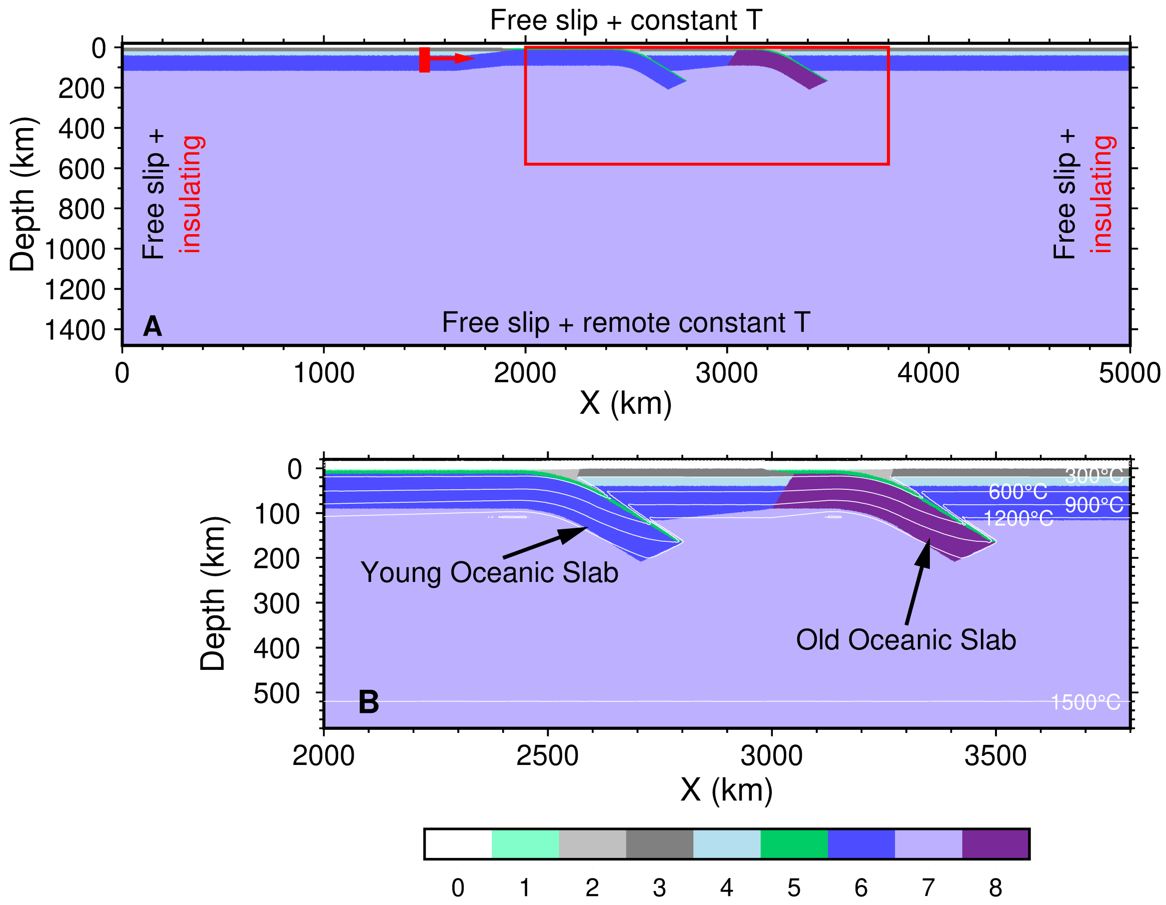

Fig. S14. Thermal-mechanical model setup.

(**A**) The entire model domain. (**B**) The zoomed-in configuration of the red rectangle in (**A**). The purple eastern slab is older and 50 kg/m3 denser than the western slab. White lines represent isotherms with an interval of 300°C. Composition code: 0 – sticky air; 1 – sea water; 2 – sediment; 3 – continental upper crust; 4 – continental lower crust; 5 – oceanic crust; 6 – lithosphere mantle; 7 – sublithospheric mantle; 8 – dense oceanic slab mantle.

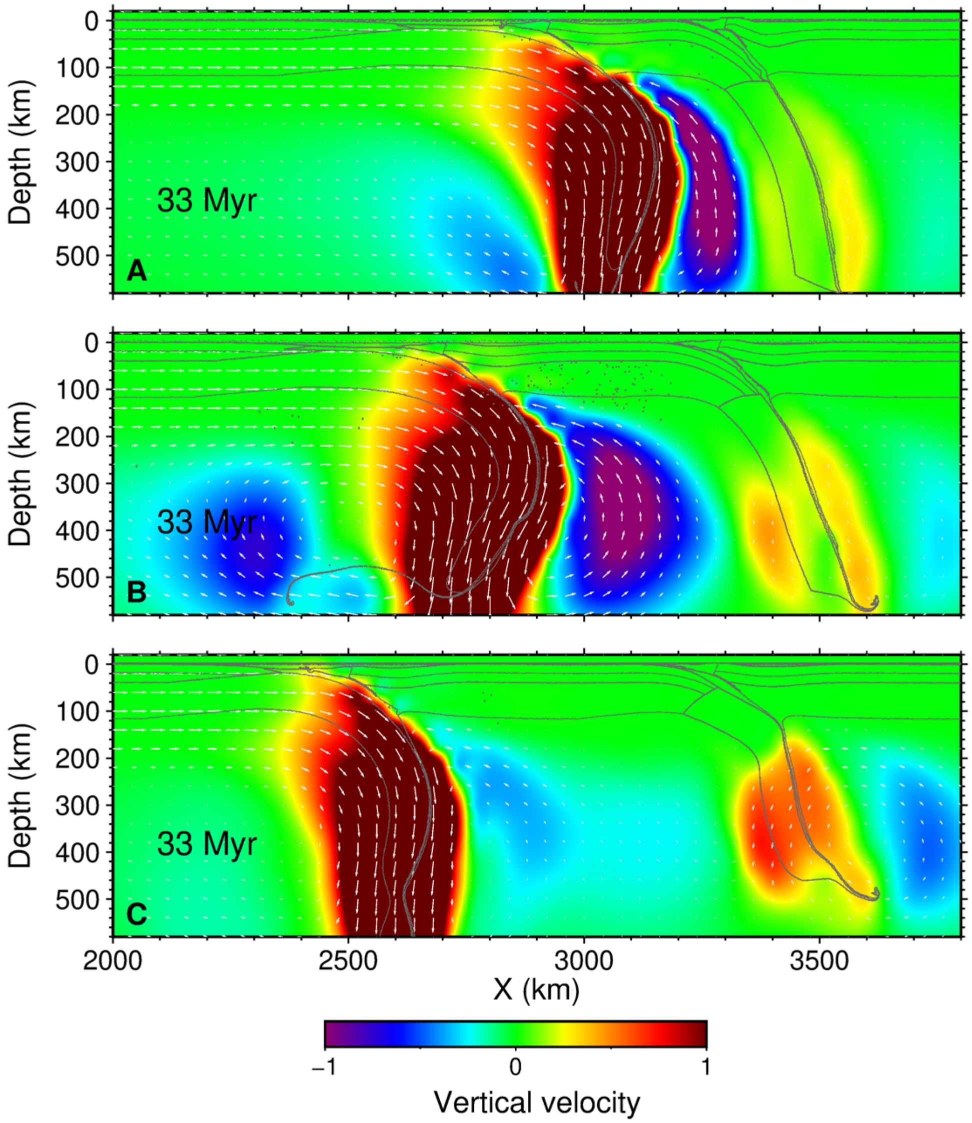

Fig. S15. Snapshots of the mantle flow field for models with different initial widths of the micro-continental block between subducting slabs.

Snapshots of the mantle flow field for the thermal-mechanical model with initial widths of ~550 km (**A**), ~750 km (**B**) and ~850 km (**C**) for the micro-continental block. The color shows the magnitude of the vertical velocity component. White arrows indicate the motion of the material. The gray lines contour the geometry of the slabs.

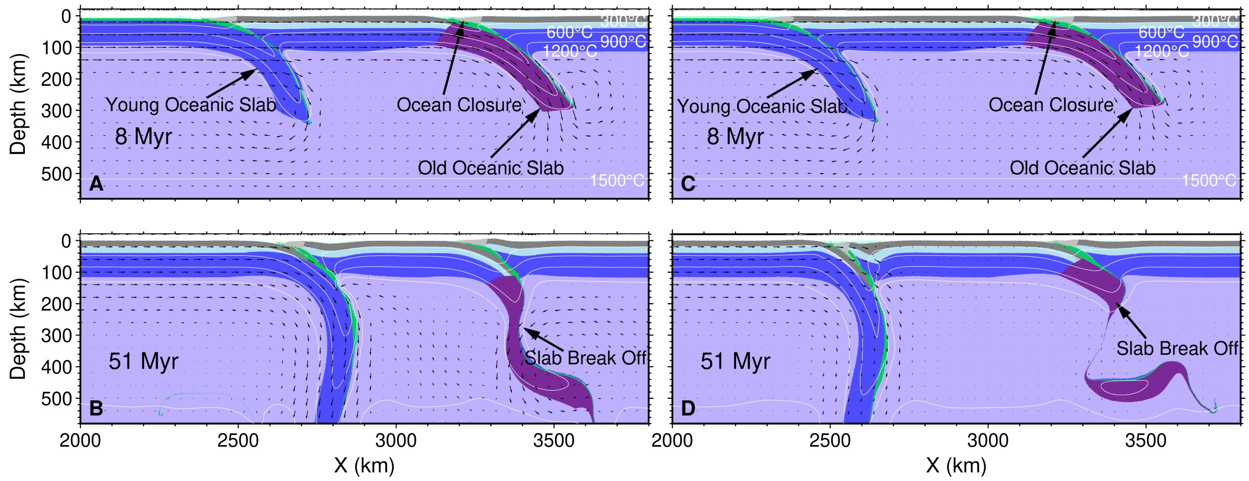

Fig. S16. Numerical modeling of double subduction for the models with different initial widths of the micro-continental block between two subduction zones.

Snapshots for the model with widths of ~750 km (**A** and **B**) and ~850 km (**C** and **D**). Black arrows indicate the velocities of material flow. White lines represent isotherms with an interval of 300°C. Time is counted from the start of the model, so slab break-off occurs ~40 Myr after the closure of the eastern ocean.

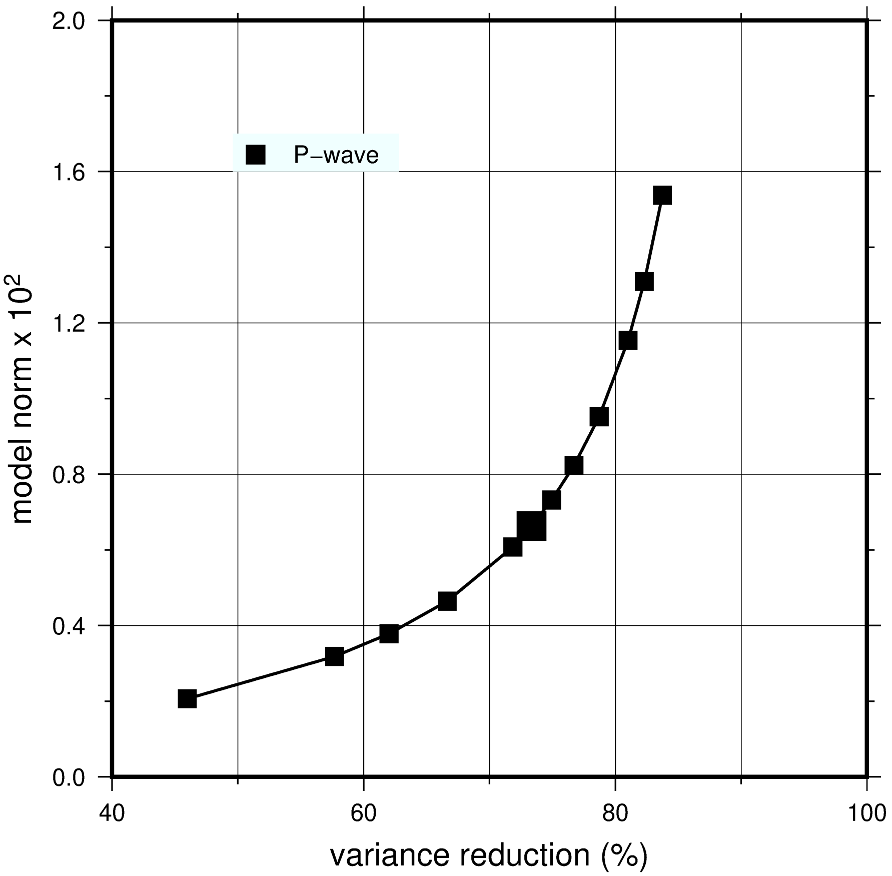

Fig. S17. Tradeoff curve for the determination of damping factor.

The larger symbol denotes the preferred value of damping used in our final models, which have a variance reduction of ~73% for the P velocity model.

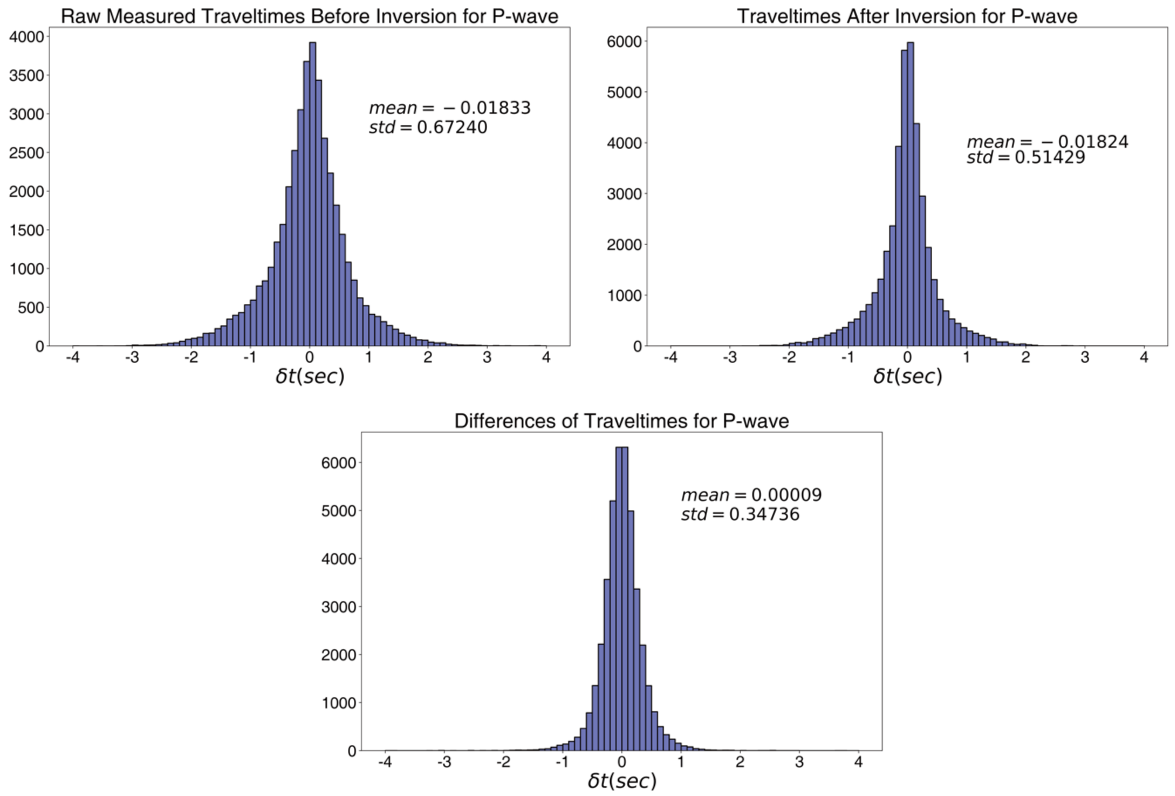

Fig. S18. Histograms of differential travel times before and after the inversion for P-wave. The x-axis represents the difference between the travel-time shifts corresponding to the selected stations and the reference stations.

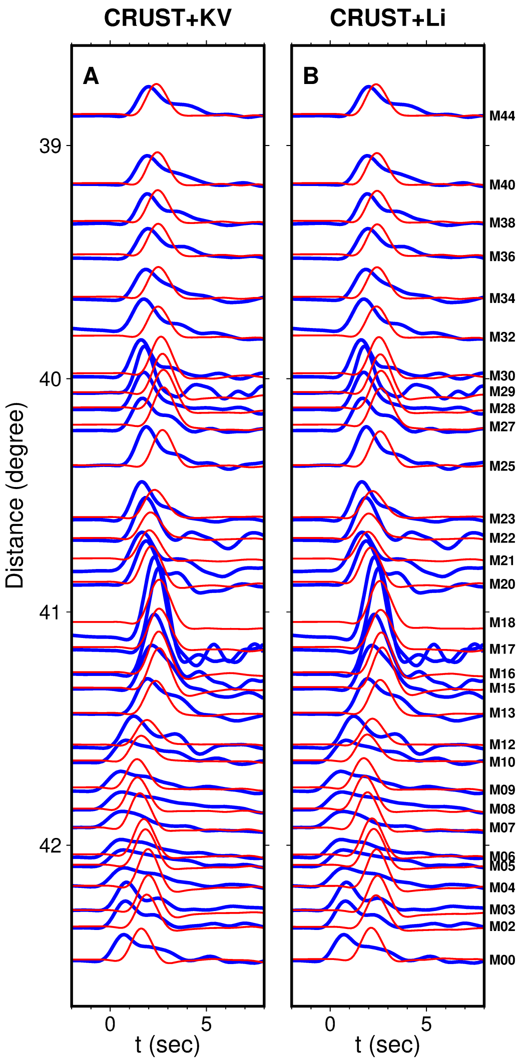

Fig. S19. Comparison of two-dimensional waveform modeling results of event 2017/09/07 based on the P-wave velocity models obtained in previous studies along the same profile.
(**A** and **B**) Comparison between observed (blue lines) and synthetic (red lines) vertical displacements generated by a model composing the crust structure (*41*) and the upper-mantle model from Koulakov (*15*) (**A**) and Li et al. (*13*) (**B**). Red and blue waveforms represent synthetic and observed vertical displacements, respectively.

Table S1. Compilation for magmatic rocks of the western magmatic belt and representative zircons from sedimentary rocks in the Central Lowland (CL) and magmatic rocks of the eastern magmatic belt in the Shan Plateau (SP).

Coordinates	Age (Ma)	Reference
A. Eastern magmatic belt in SP		
96.16331°E, 21.67786°N	44.8 ± 2.7	[26]
96.12919°E, 21.68256°N	45.9 ± 0.7	[26]
96.12917°E, 21.68278°N	44.6 ± 0.5	[26]
96.28569°E, 20.77097°N	50.0 ± 0.6	[26]
96.25967°E, 20.27803°N	71.8 ± 0.5	[26]
96.33056°E, 20.32861°N	48.0 ± 0.9	[26]
96.33056°E, 20.32861°N	53.8 ± 0.8	[26]
97.07778°E, 17.42222°N	63.3 ± 0.6	[26]
97.46667°E, 16.61667°N	70.0 ± 0.9	[26]
98.36278°E, 14.18861°N	62.3 ± 0.6	[29]
98.33583°E, 14.55111°N	69.5 ± 1.0	[29]
98.83694°E, 12.58389°N	75.3 ± 7.7	[29]
99.29389°E, 11.29583°N	50.3 ± 0.6	[29]
98.26028°E, 14.27556°N	75.6 ± 8.8	[30]
98.11833°E, 14.13750°N	64.1 ± 1.6	[30]
98.38333°E, 13.67000°N	58.5 ± 0.5	[30]
98.42028°E, 13.56806°N	58.7 ± 0.6	[30]
98.73667°E, 12.69194°N	72.1 ± 1.3	[30]
96.22472°E, 20.74111°N	71.9 ± 1.1	[49]
96.25972°E, 20.27806°N	71.1 ± 0.6	[49]
96.25917°E, 20.05806°N	55.1 ± 0.5	[49]
96.25333°E, 20.27806°N	71.1 ± 0.7	[50]
	71.1 ± 0.3	
	71.2 ± 0.4	
B. Western magmatic belt in CL		
95.11260°E, 22.78220°N	64.5 ± 0.8	[24]
95.00380°E, 22.36540°N	69.0 ± 2.6	[24]
95.59361°E, 23.70694°N	40.0 ± 0.2	[29]
C. Representative zircons from sedimentary rocks in CL		
94.29089°E, 23.19419°N	77.0 ± 2.0	[42]
	64.0 ± 1.0	
	57.0 ± 1.0	
	90.0 ± 2.0	
	53.0 ± 1.0	
94.57150°E, 22.53331°N	68.0 ± 2.0	[42]
	50.0 ± 1.0	
	89.0 ± 2.0	
	44.0 ± 1.0	
94.23103°E, 23.19967°N	63.0 ± 3.0	[42]
	87.0 ± 4.0	
	50.0 ± 3.0	
	65.0 ± 2.0	
94.22267°E, 23.20208°N	90.0 ± 2.0	[42]
	76.0 ± 2.0	
	47.0 ± 1.0	
	87.0 ± 2.0	
94.20647°E, 23.20572°N	87.0 ± 2.0	[42]
	86.0 ± 4.0	
	48.0 ± 2.0	
94.15581°E, 23.22761°N	69.0 ± 1.0	[42]
94.15211°E, 23.22408°N	69.0 ± 5.0	[42]
	81.0 ± 2.0	
94.12403°E, 23.22758°N	90.0 ± 4.0	[42]

Table S2. Information about the CMGSMO arrays used in this research.

	Station name	Latitude (°N)	Longitude (°E)	Elevation (m)	Starting time	Ending time	Sensor type	DAS type
CMGSMO I	E01	23.521105	95.664014	224	2016-06-18	2018-02-05	Trillium 120PA	Reftek 130
CMGSMO I	E02	23.242887	95.418656	163	2016-06-18	2018-02-05	Trillium 120PA	Reftek 130
CMGSMO I	E03	23.047386	94.990242	287	2016-06-19	2018-02-04	Trillium 120PA	Reftek 130
CMGSMO I	E04	22.948549	95.410381	121	2016-06-19	2017-07-31	Trillium 120PA	Reftek 130
CMGSMO I	E05	22.844243	94.810045	261	2017-03-24	2018-02-08	Trillium 120PA	Reftek 130
CMGSMO I	E06	22.487005	95.223395	261	2016-06-21	2018-02-09	Trillium 120PA	Reftek 130
CMGSMO I	E07	22.454782	94.670372	242	2016-06-23	2018-02-03	Trillium 120PA	Reftek 130
CMGSMO I	E08	22.306215	94.986445	192	2016-06-22	2018-02-03	Trillium 120PA	Reftek 130
CMGSMO I	E09	22.921449	95.645619	148	2017-08-01	2018-02-06	Trillium 120PA	Reftek 130
CMGSMO I	E10	22.955636	95.917396	120	2017-07-31	2018-02-06	Trillium 120PA	Reftek 130
CMGSMO I	E11	22.944123	96.254114	1001	2017-08-01	2018-02-08	Trillium 120PA	Reftek 130
CMGSMO I	M00	22.011337	93.174425	1465	2016-12-02	2018-01-30	Trillium 120PA	Reftek 130
CMGSMO I	M02	22.062167	93.320080	973	2016-12-12	2018-01-30	Guralp CMG3ESP.30.2000	Reftek 130
CMGSMO I	M03	22.060881	93.401781	1416	2016-12-12	2018-01-31	Guralp CMG3ESP.30.2000	Reftek 130
CMGSMO I	M04	22.106835	93.492973	1493	2016-12-11	2018-01-31	Guralp CMG3ESP.30.2000	Reftek 130
CMGSMO I	M05	22.113141	93.594765	1393	2016-12-01	2018-01-30	Trillium 120PA	Reftek 130
CMGSMO I	M06	22.098552	93.646156	1585	2016-12-11	2018-01-30	Guralp CMG3ESP.30.2000	Reftek 130
CMGSMO I	M07	22.023051	93.811493	982	2016-12-12	2017-03-25	Guralp CMG3ESP.60.2000	Reftek 130
CMGSMO I	M07	22.023051	93.811493	982	2017-03-25	2018-01-29	Guralp CMG3ESP.30.2000	Reftek 130
CMGSMO I	M08	21.985167	93.914656	629	2016-12-12	2018-01-29	Guralp CMG3ESP.30.2000	Reftek 130
CMGSMO I	M09	22.036889	93.990231	387	2016-06-20	2018-01-29	Trillium 120PA	Reftek 130
CMGSMO I	M10	22.060216	94.116539	227	2016-06-21	2016-11-15	Trillium 120PA	Reftek 130
CMGSMO I	M10	22.060216	94.116539	227	2016-12-10	2018-02-08	Guralp CMG3ESP.30.2000	Reftek 130
CMGSMO I	M11	21.997843	94.182880	321	2016-06-16	2017-01-18	Trillium 120PA	Reftek 130
CMGSMO I	M11	21.997843	94.182880	321	2017-01-18	2017-03-24	Guralp CMG3ESP.30.2000	Reftek 130
CMGSMO I	M12	21.984607	94.227102	390	2017-03-24	2018-02-09	Guralp CMG3ESP.30.2000	Reftek 130
CMGSMO I	M13	22.005340	94.373148	568	2016-06-16	2018-01-28	Trillium 120PA	Reftek 130
CMGSMO I	M15	22.031992	94.484270	268	2017-03-27	2018-01-28	Guralp CMG3ESP.30.2000	Reftek 130
CMGSMO I	M16	22.030444	94.554869	278	2016-06-16	2018-01-28	Trillium 120PA	Reftek 130
CMGSMO I	M17	22.035598	94.671355	155	2016-06-23	2016-11-18	Trillium 120PA	Reftek 130
CMGSMO I	M17	22.035598	94.671355	155	2016-12-13	2018-01-28	Guralp CMG3ESP.30.2000	Reftek 130
CMGSMO I	M18	22.089995	94.782563	126	2016-06-12	2016-11-18	Trillium 120PA	Reftek 130
CMGSMO I	M18	22.089995	94.782563	126	2016-12-13	2018-01-28	Guralp CMG3ESP.60.2000	Reftek 130
CMGSMO I	M19	22.091436	94.882356	109	2016-06-23	2016-11-08	Trillium 120PA	Reftek 130
CMGSMO I	M20	22.046989	94.983126	184	2016-06-12	2018-01-27	Trillium 120PA	Reftek 130
CMGSMO I	M21	22.100610	95.079252	173	2016-06-11	2016-11-17	Trillium 120PA	Reftek 130
CMGSMO I	M21	22.100610	95.079252	173	2017-01-12	2018-01-05	Guralp CMG3ESP.30.2000	Reftek 130
CMGSMO I	M22	22.079276	95.194328	86	2016-06-22	2016-11-17	Trillium 120PA	Reftek 130
CMGSMO I	M22	22.079276	95.194328	86	2017-01-12	2018-01-27	Guralp CMG3ESP.60.2000	Reftek 130
CMGSMO I	M23	22.090697	95.284043	171	2016-06-19	2018-02-02	Trillium 120PA	Reftek 130
CMGSMO I	M24	21.999310	95.412396	133	2016-06-11	2016-11-18	Trillium 120PA	Reftek 130
CMGSMO I	M24	21.999310	95.412396	133	2017-01-13	2017-07-21	Guralp CMG3ESP.30.2000	Reftek 130
CMGSMO I	M25	22.052570	95.552437	107	2017-07-21	2018-02-09	Guralp CMG3ESP.30.2000	Reftek 130
CMGSMO I	M26	22.044305	95.611286	99	2016-06-11	2016-11-18	Trillium 120PA	Reftek 130
CMGSMO I	M26	22.044305	95.611286	99	2017-01-13	2017-07-22	Guralp CMG3ESP.60.2000	Reftek 130
CMGSMO I	M27	22.044377	95.746330	188	2017-07-22	2018-02-09	Guralp CMG3ESP.60.2000	Reftek 130
CMGSMO I	M28	22.019879	95.842070	118	2016-06-11	2018-01-29	Trillium 120PA	Reftek 130
CMGSMO I	M29	21.999740	95.930664	106	2016-06-23	2016-11-18	Trillium 120PA	Reftek 130
CMGSMO I	M29	21.999740	95.930664	106	2017-01-15	2018-01-29	Guralp CMG3ESP.60.2000	Reftek 130
CMGSMO I	M30	22.009748	96.003992	96	2016-08-30	2016-11-19	Trillium 120PA	Reftek 130
CMGSMO I	M30	22.009748	96.003992	96	2017-01-14	2018-01-29	Guralp CMG3ESP.30.2000	Reftek 130
CMGSMO I	M31	22.013961	96.106230	172	2016-06-20	2016-11-06	Trillium 120PA	Reftek 130
CMGSMO I	M32	21.999616	96.194425	112	2016-06-07	2016-11-19	Trillium 120PA	Reftek 130
CMGSMO I	M32	21.999616	96.194425	112	2017-01-14	2018-02-01	Guralp CMG3ESP.30.2000	Reftek 130
CMGSMO I	M34	21.974041	96.390583	892	2016-06-20	2018-01-30	Trillium 120PA	Reftek 130
CMGSMO I	M36	22.032680	96.573429	1059	2016-06-10	2016-11-21	Trillium 120PA	Reftek 130
CMGSMO I	M36	22.032680	96.573429	1059	2017-01-16	2018-01-30	Guralp CMG3ESP.30.2000	Reftek 130
CMGSMO I	M38	22.201324	96.680617	686	2016-06-08	2018-01-31	Trillium 120PA	Reftek 130
CMGSMO I	M40	22.313176	96.827863	900	2016-06-08	2016-11-20	Trillium 120PA	Reftek 130
CMGSMO I	M40	22.313176	96.827863	900	2017-01-16	2018-01-31	Guralp CMG3ESP.30.2000	Reftek 130
CMGSMO I	M42	22.349790	96.939501	961	2016-06-26	2017-07-31	Trillium 120PA	Reftek 130
CMGSMO I	M44	22.552080	97.083504	685	2016-06-09	2016-11-20	Trillium 120PA	Reftek 130
CMGSMO I	M44	22.552080	97.083504	685	2017-01-16	2018-02-01	Guralp CMG3ESP.30.2000	Reftek 130
CMGSMO I	M46	22.601181	97.313942	513	2016-06-26	2017-07-31	Trillium 120PA	Reftek 130
CMGSMO I	N01	23.889773	93.531027	828	2017-01-30	2018-02-04	Trillium 120PA	Reftek 130
CMGSMO I	N02	23.837218	94.148093	194	2016-06-13	2017-09-13	Trillium 120PA	Reftek 130
CMGSMO I	N03	23.833004	94.565248	124	2016-12-08	2018-02-07	Trillium 120PA	Reftek 130
CMGSMO I	N04	23.599806	93.730033	1298	2016-11-26	2018-02-05	Trillium 120PA	Reftek 130
CMGSMO I	N05	23.522401	94.089435	188	2016-06-13	2018-02-06	Trillium 120PA	Reftek 130
CMGSMO I	N06	23.609372	94.414497	135	2016-06-14	2018-02-07	Trillium 120PA	Reftek 130
CMGSMO I	N07	23.347400	93.389600	989	2016-11-27	2018-02-03	Trillium 120PA	Reftek 130
CMGSMO I	N08	23.219643	93.809777	2031	2016-12-09	2018-02-05	Trillium 120PA	Reftek 130
CMGSMO I	N09	23.292468	94.113507	146	2016-06-13	2018-02-06	Trillium 120PA	Reftek 130
CMGSMO I	N10	23.202729	94.609811	325	2016-06-14	2018-02-07	Trillium 120PA	Reftek 130
CMGSMO I	N11	22.935017	93.704795	1041	2016-11-25	2018-02-03	Trillium 120PA	Reftek 130
CMGSMO I	N12	22.810082	93.972031	210	2016-06-15	2018-02-01	Trillium 120PA	Reftek 130
CMGSMO I	N13	22.950781	94.315489	108	2016-11-14	2018-02-07	Trillium 120PA	Reftek 130
CMGSMO I	N14	22.688526	93.430636	1547	2016-11-24	2018-02-02	Trillium 120PA	Reftek 130
CMGSMO I	N15	22.477392	93.802637	1223	2016-11-16	2018-02-02	Trillium 120PA	Reftek 130
CMGSMO I	N16	22.427897	94.072317	209	2016-06-15	2018-02-01	Trillium 120PA	Reftek 130
CMGSMO I	N17	22.708239	94.398153	150	2016-12-14	2018-02-07	Trillium 120PA	Reftek 130
CMGSMO I	N18	23.395994	93.675583	1505	2016-11-28	2018-02-04	Trillium 120PA	Reftek 130
CMGSMO I	N19	23.049782	93.469445	1246	2016-11-26	2018-02-03	Trillium 120PA	Reftek 130
CMGSMO I	S01	21.689242	93.469505	1186	2016-12-05	2018-01-31	Trillium 120PA	Reftek 130
CMGSMO I	S02	21.667696	94.137149	352	2016-06-17	2018-02-01	Trillium 120PA	Reftek 130

Table S2. (*continued*)

	Station name	Latitude (°N)	Longitude (°E)	Elevation (m)	Starting time	Ending time	Sensor type	DAS type
CMGSMO II	C26	21.500230	96.264990	136	2019-01-17	2020-07-30	Trillium 120PA	Taurus
CMGSMO II	C29	20.277270	96.241470	185	2019-02-21	2020-07-31	Trillium 120PA	Taurus
CMGSMO II	C33	21.642610	96.921080	722	2019-02-22	2020-08-15	Trillium 120PA	Taurus
CMGSMO II	C34	21.525670	97.549750	1148	2019-03-03	2020-08-10	Trillium 120PA	Taurus
CMGSMO II	C35	21.351810	98.069700	1148	2019-03-05	2020-08-11	Trillium 120PA	Taurus
CMGSMO II	C36	21.209360	98.783870	743	2019-03-08	2020-08-12	Trillium 120PA	Taurus
CMGSMO II	C37	21.915590	98.366660	753	2019-03-10	2020-08-10	Trillium 120PA	Taurus
CMGSMO II	C38	20.973790	96.641180	1542	2019-02-25	2020-08-16	Trillium 120PA	Taurus
CMGSMO II	C39	20.918470	97.292530	1558	2019-02-23	2020-08-08	Trillium 120PA	Taurus
CMGSMO II	C40	20.652230	97.955760	992	2019-03-12	2020-08-13	Trillium 120PA	Taurus
CMGSMO II	C41	20.329000	98.859410	585	2019-03-05	2020-08-15	Trillium 120 Posthole	Centaur
CMGSMO II	C42	20.477900	96.712660	1542	2019-02-21	2020-08-14	Trillium 120PA	Taurus
CMGSMO II	C43	20.152030	97.295610	1115	2019-02-23	2020-08-08	Trillium 120PA	Taurus
CMGSMO II	C44	20.296380	98.345970	703	2019-03-13	2020-08-15	Trillium 120PA	Taurus

REFERENCES AND NOTES

1. J. F. Dewey, J. M. Bird, Mountain belts and the new global tectonics. *J. Geophys. Res.* **75**, 2625–2647 (1970).

2. D. J. J. Van Hinsbergen, P. C. Lippert, G. Dupont-Nivert, N. McQuarrie, P. V. Doubrovine, W. Spakman, T. H. Torsvik, Greater India Basin hypothesis and a two-stage Cenozoic collision between India and Asia. *Proc. Natl. Acad. Sci. U.S.A.* **109**, 7659–7664 (2012).

3. S. C. Cande, D. R. Stegman, Indian and African plate motions driven by the push force of the Réunion plume head. *Nature* **475**, 47–52 (2011).

4. L. T. White, G. S. Lister, The collision of India with Asia. *J. Geodyn.* **56-57**, 7–17 (2012).

5. P. Kumar, X. Yuan, M. R. Kumar, R. Kind, X. Li, R. K. Chadha, The rapid drift of the Indian tectonic plate. *Nature* **449**, 894–897 (2007).

6. O. Jagoutz, L. Royden, A. F. Holt, T. W. Becker, Anomalously fast convergence of India and Eurasia caused by double subduction. *Nat. Geosci.* **8**, 475–478 (2015).

7. P. Bouilhol, O. Jagoutz, J. M. Hanchar, F. O. Dudas, Dating the India-Eurasia collision through arc magmatic records. *Earth Planet. Sci. Lett.* **366**, 163–175 (2013).

8. J. Westerweel, P. Roperch, A. Licht, G. Dupont-Nivet, Z. Win, F. Poblete, G. Ruffet, H. H. Swe, M. K. Thi, D. W. Aung, Burma Terrane part of the Trans-Tethyan arc during collision with India according to palaeomagnetic data. *Nat. Geosci.* **12**, 863–868 (2019).

9. R. Van Der Voo, W. Spakman, H. Bijwaard, Tethyan subducted slabs under India. *Earth Planet. Sci. Lett.* **171**, 7–20 (1999).

10. X. Liang, Y. Chen, X. Tian, Y. J. Chen, J. Ni, A. Gallegos, S. L. Klemperer, M. Wang, T. Xu, C. Sun, S. Si, H. Lan, J. Teng, 3D imaging of subducting and fragmenting Indian continental lithosphere beneath southern and central Tibet using body-wave finite-frequency tomography. *Earth Planet. Sci. Lett.* **443**, 162–175 (2016).

11. S. K. Kufner, N. Kakar, M. Bezada, W. Bloch, S. Metzger, X. Yuan, J. Mechie, L. Ratschbacher, S. Murodkulov, Z. Deng, B. Schurr, The Hindu Kush slab break-off as revealed by deep structure and crustal deformation. *Nat. Commun.* **12**, 1685 (2021).

12. T. Zheng, Y. He, L. Ding, M. Jiang, Y. Ai, C. T. Mon, G. Hou, K. Sein, M. Thant, Direct structural evidence of Indian continental subduction beneath Myanmar. *Nat. Commun.* **11**, 1944 (2020).

13. C. Li, R. D. van der Hilst, A. S. Meltzer, E. R. Engdahl, Subduction of the Indian lithosphere beneath the Tibetan Plateau and Burma. *Earth Planet. Sci. Lett.* **274**, 157–168 (2008).

14. A. Replumaz, A. M. Negredo, S. Guillot, A. Villaseñor, Multiple episodes of continental subduction during India/Asia convergence: Insight from seismic tomography and tectonic reconstruction. *Tectonophysics* **483**, 125–134 (2010).

15. I. Koulakov, High-frequency P and S velocity anomalies in the upper mantle beneath Asia from inversion of worldwide traveltime data. *J. Geophys. Res. Solid Earth* **116**, (2011).

16. R. A. Sloan, J. R. Elliott, M. P. Searle, C. K. Morley, Chapter 2: Active tectonics of Myanmar and the Andaman Sea. *Geol. Soc. Mem.* **48**, 19–52 (2017).

17. B. Wan, F. Wu, L. Chen, L. Liang, X. Liang, W. Xiao, R. Zhu, Cyclical one-way continental rupture-drift in the Tethyan evolution: Subduction-driven plate tectonics. *Sci. China Earth Sci.* **62**, 2005–2016 (2019).

18. C. Z. Liu, S. L. Chung, F. Y. Wu, C. Zhang, Y. Xu, J. G. Wang, Y. Chen, S. Guo, Tethyan suturing in Southeast Asia: Zircon U-Pb and Hf-O isotopic constraints from Myanmar ophiolites. *Geology* **44**, 311–314 (2016).

19. J. Zhang, W. Xiao, B. F. Windley, J. Wakabayashi, F. Cai, K. Sein, H. Wu, S. Naing, Multiple alternating forearc- and backarc-ward migration of magmatism in the Indo-Myanmar Orogenic Belt since the Jurassic: Documentation of the orogenic architecture of eastern Neotethys in SE Asia. *Earth Sci. Rev.* **185**, 704–731 (2018).

20. M. E. Barley, A. L. Pickard, K. Zaw, P. Rak, M. G. Doyle, Jurassic to Miocene magmatism and metamorphism in the Mogok metamorphic belt and the India-Eurasia collision in Myanmar. *Tectonics* **22**, 1019 (2003).

21. M. P. Searle, S. R. Noble, J. M. Cottle, D. J. Waters, A. H. G. Mitchell, T. Hlaing, M. S. A. Horstwood, Tectonic evolution of the Mogok metamorphic belt, Burma (Myanmar) constrained by U-Th-Pb dating of metamorphic and magmatic rocks. *Tectonics* **26**, TC3014 (2007).

22. M. P. Searle, J. M. Garber, B. R. Hacker, K. Htun, N. J. Gardiner, D. J. Waters, L. J. Robb, Timing of syenite-charnockite magmatism and ruby and sapphire metamorphism in the mogok valley region, Myanmar. *Tectonics* **39**, e2019TC005998 (2020).

23. K. Khin, K. Zaw, L. T. Aung, Chapter 4: Geological and tectonic evolution of the Indo-Myanmar Ranges (IMR) in the Myanmar region. *Geol. Soc. Mem.* **48**, 65–79 (2017).

24. P. Zhang, L. Mei, X. Hu, R. Li, L. Wu, Z. Zhou, H. Qiu, Structures, uplift, and magmatism of the Western Myanmar Arc: Constraints to mid-Cretaceous-Paleogene tectonic evolution of the western Myanmar continental margin. *Gondw. Res.* **52**, 18–38 (2017).

25. K. Zaw, W. Swe, A. J. Barber, M. J. Crow, Y. Y. Nwe, Chapter 1: Introduction to the geology of Myanmar. *Geol. Soc. Mem.* **48**, 1–17 (2017).

26. A. Mitchell, S. L. Chung, T. Oo, T. H. Lin, C. H. Hung, Zircon U-Pb ages in Myanmar: Magmatic-metamorphic events and the closure of a neo-Tethys ocean? *J. Asian Earth Sci.* **56**, 1–23 (2012).

27. R. Li, L. Mei, G. Zhu, R. Zhao, X. Xu, H. Zhao, P. Zhang, Y. Yin, Y. Ma, Late mesozoic to cenozoic tectonic events in volcanic arc, West Burma Block: Evidences from U-Pb zircon dating and apatite fission track data of granitoids. *J. Earth Sci.* **24**, 553–568 (2013).

28. H. Y. Lee, S. L. Chung, H. M. Yang, Late Cenozoic volcanism in central Myanmar: Geochemical characteristics and geodynamic significance. *Lithos* **245**, 174–190 (2016).

29. N. J. Gardiner, L. J. Robb, C. K. Morley, M. P. Searle, P. A. Cawood, M. J. Whitehouse, C. L. Kirkland, N. M. W. Roberts, T. A. Myint, The tectonic and metallogenic framework of Myanmar: A Tethyan mineral system. *Ore Geol. Rev.* **79**, 26–45 (2016).

30. N. J. Gardiner, C. J. Hawkesworth, L. J. Robb, M. J. Whitehouse, N. M. W. Roberts, C. L. Kirkland,N. J. Evans, Contrasting granite metallogeny through the zircon record: A case study from Myanmar. *Sci. Rep.* **7**, 748 (2017).

31. T. H. Lin, A. H. G. Mitchell, S. L. Chung, X. Tan, T. Oo, J. T. Tang, F. Y. Wu, Two parallel magmatic belts with contrasting isotopic characteristics from southern tibet to myanmar: Zircon U–Pb and Hf isotopic constraints. *J. Geol. Soc. London.* **176**, 574–587 (2019).

32. J. X. Li, W. M. Fan, L. Y. Zhang, L. Ding, Y. L. Sun, T. P. Peng, F. L. Cai, Q. Y. Guan, K. Sein, Subduction of Indian continental lithosphere constrained by Eocene-Oligocene magmatism in northern Myanmar. *Lithos* **348–349**, 105211 (2019).

33. J. X. Li, W. M. Fan, L. Y. Zhang, T. P. Peng, Y. L. Sun, L. Ding, F. L. Cai, K. Sein, Prolonged Neo-Tethyan magmatic arc in Myanmar: Evidence from geochemistry and Sr–Nd–Hf isotopes of Cretaceous mafic–felsic intrusions in the Banmauk–Kawlin area. *Int. J. Earth Sci.* **109**, 649–668 (2020).

34. T. Maurin, C. Rangin, Structure and kinematics of the Indo-Burmese Wedge: Recent and fast growth of the outer wedge. *Tectonics* **28**, 1–21 (2009).

35. C. T. Mon, X. Gong, Y. Wen, M. Jiang, Q. F. Chen, M. Zhang, G. Hou, M. Thant, K. Sein, Y. He, Insight into major active faults in central Myanmar and the related geodynamic sources. *Geophys. Res. Lett.* **47**, e2019GL086236 (2020).

36. S. H. Hung, Y. Shen, L. Y. Chiao, Imaging seismic velocity structure beneath the Iceland hot spot: A finite frequency approach. *J. Geophys. Res. Solid Earth* **109**, B08305 (2004).

37. Y. Tang, M. Obayashi, F. Niu, S. P. Grand, Y. J. Chen, H. Kawakatsu, S. Tanaka, J. Ning, Changbaishan volcanism in northeast China linked to subduction-induced mantle upwelling. *Nat. Geosci.* **7**, 470–475 (2014).

38. K. Yuan, B. Romanowicz, Seismic evidence for partial melting at the root of major hot spot plumes. *Science* **357**, 393–397 (2017).

39. D. Li, D. Helmberger, R. W. Clayton, D. Sun, Global synthetic seismograms using a 2-D finite-difference method. *Geophys. J. Int.* **197**, 1166–1183 (2014).

40. J. Ni, M. Guzmán-Speziale, M. Bevis, W. E. Holt, T. C. Wallace, W. R. Seager, Accretionary tectonics of Burma and the three-dimensional geometry of the Burma subduction zone. *Geology* **17**, 68–71 (1989).

41. G. Zhang, Y. He, Y. Ai, M. Jiang, C. T. Mon, G. Hou, M. Thant, K. Sein, Indian continental lithosphere and related volcanism beneath Myanmar: Constraints from local earthquake tomography. *Earth Planet. Sci. Lett.* **567**, 116987 (2021).

42. J. G. Wang, F. Y. Wu, X. C. Tan, C. Z. Liu, Magmatic evolution of the Western Myanmar Arc documented by U-Pb and Hf isotopes in detrital zircon. *Tectonophysics* **612–613**, 97–105 (2014).

43. A. Replumaz, H. Kárason, R. D. van der Hilst, J. Besse, P. Tapponnier, 4-D evolution of SE Asia's mantle from geological reconstructions and seismic tomography. *Earth Planet. Sci. Lett.* **221**, 103–115 (2004).

44. X. Bao, D. W. Eaton, B. Guest, Plateau uplift in western Canada caused by lithospheric delamination along a craton edge. *Nat. Geosci.* **7**, 830–833 (2014).

45. P. Agard, J. Omrani, L. Jolivet, H. Whitechurch, B. Vrielynck, W. Spakman, P. Monie, B. Meyer, R. Wortel, Zagros orogeny: A subduction-dominated process. *Geol. Mag.* **148**, 692–725 (2011).

46. F. Cammarano, S. Goes, P. Vacher, D. Giardini, Inferring upper-mantle temperatures from seismic velocities. *Phys. Earth Planet. Inter.* **138**, 197–222 (2003).

47. I. Metcalfe, Gondwana dispersion and Asian accretion: Tectonic and palaeogeographic evolution of eastern Tethys. *J. Asian Earth Sci.* **66**, 1–33 (2013).

48. R. Freeburn, P. Bouilhol, B. Maunder, V. Magni, J. van Hunen, Numerical models of the magmatic processes induced by slab breakoff. *Earth Planet. Sci. Lett.* **478**, 203–213 (2017).

49. N. J. Gardiner, M. P. Searle, C. K. Morley, L. J. Robb, M. J. Whitehouse, N. M. W. Roberts, C. L. Kirkland, C. J. Spencer, The crustal architecture of Myanmar imaged through zircon U-Pb, Lu-Hf and O isotopes: Tectonic and metallogenic implications. *Gondw. Res.* **62**, 27–60 (2018).

50. J. X. Li, W. M. Fan, L. Y. Zhang, L. Ding, Y. H. Yue, J. Xie, F. L. Cai, K. Sein, B-rich melt immiscibility in late cretaceous Nattaung granite, Myanmar: Implication by composition and B isotope in tourmaline. *Lithos* **356–357**, 105380 (2020).

51. J. C. Aitchison, Badengzhu, A. M. Davis, J. Liu, H. Luo, J. G. Malpas, I. R. C. McDermid, H. Wu, S. V. Ziabrev, M. F. Zhou, Remnants of a Cretaceous intra-oceanic subduction system within the Yarlung-Zangbo suture (southern Tibet). *Earth Planet. Sci. Lett.* **183**, 231–244 (2000).

52. R. Hébert, R. Bezard, C. Guilmette, J. Dostal, C. S. Wang, Z. F. Liu, The Indus-Yarlung Zangbo ophiolites from Nanga Parbat to Namche Barwa syntaxes, southern Tibet: First synthesis of petrology, geochemistry, and geochronology with incidences on geodynamic reconstructions of Neo-Tethys. *Gondw. Res.* **22**, 377–397 (2012).

53. J. Yuan, Z. Yang, C. Deng, W. Krijgsman, X. Hu, S. Li, Z. Shen, H. Qin, W. An, H. He, L. Ding, Z. Guo, R. Zhu, Rapid drift of the Tethyan Himalaya terrane before two-stage India-Asia collision. *Natl. Sci. Rev.* **8**, 1–13 (2020).

54. C. R. Martin, O. Jagoutz, R. Upadhyay, L. H. Royden, M. P. Eddy, E. Bailey, C. Nichols, B. P. Weiss, Paleocene latitude of the Kohistan–Ladakh arc indicates multistage India–Eurasia collision. *Proc. Natl. Acad. Sci. U.S.A.* **117**, 29487–29494 (2020).

55. X. F. Zheng, Z. X. Yao, J. H. Liang, J. Zheng, The role played and opportunities provided by IGP DMC of China National seismic network in wenchuan earthquake disaster relief and researches. *Bull. Seismol. Soc. Am.* **100**, 2866–2872 (2010).

56. Z. Ding, Z.Wu, Advances of ChinArray program, in *Proceedings of the AGU Fall Meeting* (ARCUS, 2013).

57. X. Lou, S. Van Der Lee, S. Lloyd, AIMBAT: A python/matplotlib tool for measuring teleseismic arrival times. *Seismol. Res. Lett.* **84**, 85–93 (2013).

58. F. A. Dahlen, S. H. Hung, G. Nolet, Fréchet kernels for finite-frequency traveltimes-I. Theory. *Geophys. J. Int.* **141**, 157–174 (2000).

59. S. H. Hung, F. A. Dahlen, G. Nolet, Fréchet kernels for finite-frequency traveltimes-II. Examples. *Examples. Geophys. J. Int.* **141**, 175–203 (2000).

60. C. C. Paige, M. A. Saunders, LSQR: An algorithm for sparse linear equations and sparse least squares. *ACM Trans. Math. Softw.* **8**, 43–71 (1982).

61. T. Yang, Y. Shen, Frequency-dependent crustal correction for finite-frequency seismic tomography. *Bull. Seismol. Soc. Am.* **96**, 2441–2448 (2006).

62. G. Laske, G. Masters, Z. Ma, M. Pasyanos, Update on CRUST1.0 - A 1-degree global model of Earth's crust. *EGU Gen. Assem.* **15**, 2658 (2013).

63. Y. Wang, Y. He, G. Lu, L. Wen, Seismic, thermal and compositional structures of the stagnant slab in the mantle transition zone beneath southeastern China. *Tectonophysics* **775**, 228208 (2020).

64. D. J. Weidner, Y. Wang, Chemical- and Clapeyron-induced buoyancy at the 660 km discontinuity. *J. Geophys. Res.* **103**, 7431–7441 (1998).

65. T. V. Gerya, D. A. Yuen, Characteristics-based marker-in-cell method with conservative finite-differences schemes for modeling geological flows with strongly variable transport properties. *Phys. Earth Planet. Inter.* **140**, 293–318 (2003).

66. T. V. Gerya, *Introduction to Numerical Geodynamic Modelling* (Cambridge University Press, 2010).

67. J. P. Burg, T. V. Gerya, The role of viscous heating in Barrovian metamorphism of collisional orogens: Thermomechanical models and application to the Lepontine Dome in the Central Alps. *J. Metam. Geol.* **23**, 75–95 (2005).

68. H. Schmeling, A. Y. Babeyko, A. Enns, C. Faccenna, F. Funiciello, T. Gerya, G. J. Golabek, S. Grigull, B. J. P. Kaus, G. Morra, S. M. Schmalholz, J. Van Hunen, A benchmark comparison of spontaneous subduction models—Towards a free surface. *Phys. Earth Planet. Inter.* **171**, 198–223 (2008).

69. A. Licht, G. Dupont-Nivet, Z. Win, H. H. Swe, M. Kaythi, P. Roperch, T. Ugrai, V. Littell, D. Park, J. Westerweel, D. Jones, F. Poblete, D. W. Aung, H. Huang, C. Hoorn, K. Sein, Paleogene evolution of the Burmese forearc basin and implications for the history of India-Asia convergence. *Bull. Geol. Soc. Am.* **131**, 730–748 (2019).

70. X. Wang, S. Wei, Y. Wang, P. M. Maung, J. Hubbard, P. Banerjee, B. S. Huang, K. M. Oo, T. Bodin, A. Foster, R. Almeida, A 3-D shear wave velocity model for Myanmar region. *J. Geophys. Res. Solid Earth* **124**, 504–526 (2019).

71. G. P. Hayes, G. L. Moore, D. E. Portner, M. Hearne, H. Flamme, M. Furtney, G. M. Smoczyk, Slab2, a comprehensive subduction zone geometry model. *Science* **362**, 58–61 (2018).

72. A. Licht, Z. Win, J. Westerweel, N. Cogné, C. K. Morley, S. Chantraprasert, F. Poblete, T. Ugrai, B. Nelson, D. W. Aung, G. Dupont-Nivet, Magmatic history of central Myanmar and implications for the evolution of the Burma Terrane. *Gondw. Res.* **87**, 303–319 (2020).

73. B. L. N. Kennett, E. R. Engdahl, Traveltimes for global earthquake location and phase identification. *Geophys. J. Int.* **105**, 429–465 (1991).
